# CRISPR/Cas9 mediated mutation of the *mtnr1a* melatonin receptor gene causes rod photoreceptor degeneration in developing *Xenopus tropicalis*

**DOI:** 10.1038/s41598-020-70735-2

**Published:** 2020-08-13

**Authors:** Allan F. Wiechmann, Teryn A. Martin, Marko E. Horb

**Affiliations:** 1 Department of Cell Biology, University of Oklahoma Health Sciences Center, Oklahoma City, OK USA; 2 Department of Ophthalmology, University of Oklahoma Health Sciences Center, Oklahoma City, OK USA; 3 Marine Biological laboratory, Woods Hole, MA USA

**Keywords:** Circadian rhythms, Eye diseases

## Abstract

Nighttime surges in melatonin levels activate melatonin receptors, which synchronize cellular activities with the natural light/dark cycle. Melatonin receptors are expressed in several cell types in the retina, including the photon-sensitive rods and cones. Previous studies suggest that long-term photoreceptor survival and retinal health is in part reliant on melatonin orchestration of circadian homeostatic activities. This scenario would accordingly envisage that disruption of melatonin receptor signaling is detrimental to photoreceptor health. Using in vivo CRISPR/Cas9 genomic editing, we discovered that a small deletion mutation of the Mel1a melatonin receptor *(mtnr1a)* gene causes a loss of rod photoreceptors in retinas of developing *Xenopus tropicalis* heterozygous, but not homozygous mutant tadpoles. Cones were relatively spared from degeneration, and the rod loss phenotype was not obvious after metamorphosis. Localization of Mel1a receptor protein appeared to be about the same in wild type and mutant retinas, suggesting that the mutant protein is expressed at some level in mutant retinal cells. The severe impact on early rod photoreceptor viability may signify a previously underestimated critical role in circadian influences on long-term retinal health and preservation of sight. These data offer evidence that disturbance of homeostatic, circadian signaling, conveyed through a mutated melatonin receptor, is incompatible with rod photoreceptor survival.

## Introduction

Circadian (daily) rhythms synchronize to the natural solar cycle via photoreceptive cells in the retina of the eye that detect radiant light energy and convey that information to inner retinal cells, some of which project to the brain. Cyclic oscillations of intracellular activity occur in many cells of the body, and are especially robust in retinal cells since they need to anticipate the 12 magnitudes in ambient light intensity changes that can occur over a 24-h period^1^. The key circadian signaling molecule of darkness, melatonin, is produced by retinal photoreceptors at nighttime to drive circadian events in the eye and is secreted into the cerebrospinal fluid and general circulation from the pineal gland^2^. Melatonin binds to integral membrane G protein-coupled receptors (GPCRs) expressed in target cells throughout the body to harmonize cellular activities with the predictable 24-h day/night cycle^3^. Melatonin receptor subtypes are designated as Mel1a, Mel1b, and Mel1c in amphibians, fish and birds^4^, and as MT1, MT2, and GPR50 (which does not bind melatonin) in mammals, respectively^3^.

One primary function of melatonin in the retina is to enhance visual sensitivity to light at nighttime^5^. If the solar light cycle is disengaged from circadian cellular readiness, the metabolic imbalance imposed on dark-adapted photoreceptors by radiant light energy causes cell damage^6^. The increased vulnerability of photoreceptors to light damage during subjective night^7^ is consistent with reports that melatonin receptor activation exacerbates light-induced rod cell death^8,9^. Together, these studies support the overarching hypothesis that unabated activation of melatonin receptors is detrimental to long-term photoreceptor health. Another key role of retinal melatonin is to synchronize the daily shedding of photoreceptor outer segment distal tips and their phagocytosis by the adjacent retinal pigment epithelial (RPE) cells^10^. The circadian turnover of rod photoreceptor outer segments (ROS) occurs in the early morning in coordination with light onset^11,12^, and is essential for long-term photoreceptor health^13^. An understanding of the molecular mechanisms by which melatonin coordinates cyclic ROS shedding and RPE phagocytosis is incomplete.

Historically, *Xenopus laevis* has been a key animal model in understanding the circadian synthesis and functions of melatonin in the retina. Melatonin is synthesized by *Xenopus* photoreceptors at night^14–16^, due to clock-regulated synthesizing enzymes^17^. Cone photoreceptor inner segments elongate at night and contract during the day under the influence of melatonin as a dark-adaptive response in *Xenopus*^18,19^. The daily shedding of ROS each morning and their subsequent phagocytosis by the RPE is influenced by melatonin^10^. In *Xenopus* retina, dopamine is synthesized by amacrine cells during the daytime, which suppresses melatonin synthesis^20^. Conversely, melatonin production at night inhibits the release of dopamine from amacrine cells^15,21^. Dopamine shifts the balance of inputs to horizontal cells from rods to cones, which enhances cone input during the day^21^. Melatonin is considered to be both an input signal and an output of the circadian clock in *Xenopus* photoreceptors^22^.

Prior to the retinal circadian studies utilizing the *Xenopus* model, an early study on anuran *(Rana pipiens)* tadpoles provided the first evidence that the pineal gland secretes a compound that causes perinuclear aggregation of melanosomes in dermal melanophores^23^. The discovery by Lerner et al*.*^24^ of melatonin extracted of bovine pineal gland was facilitated by an in vitro pigment dispersion assay in explant cultures of frog (*R. pipiens*) skin. Further, the early studies by Bagnara and co-workers with *X. laevis* tadpoles confirmed melatonin as the skin-blanching signal of the pineal gland in vivo^25–27^.

**Figure 1:**
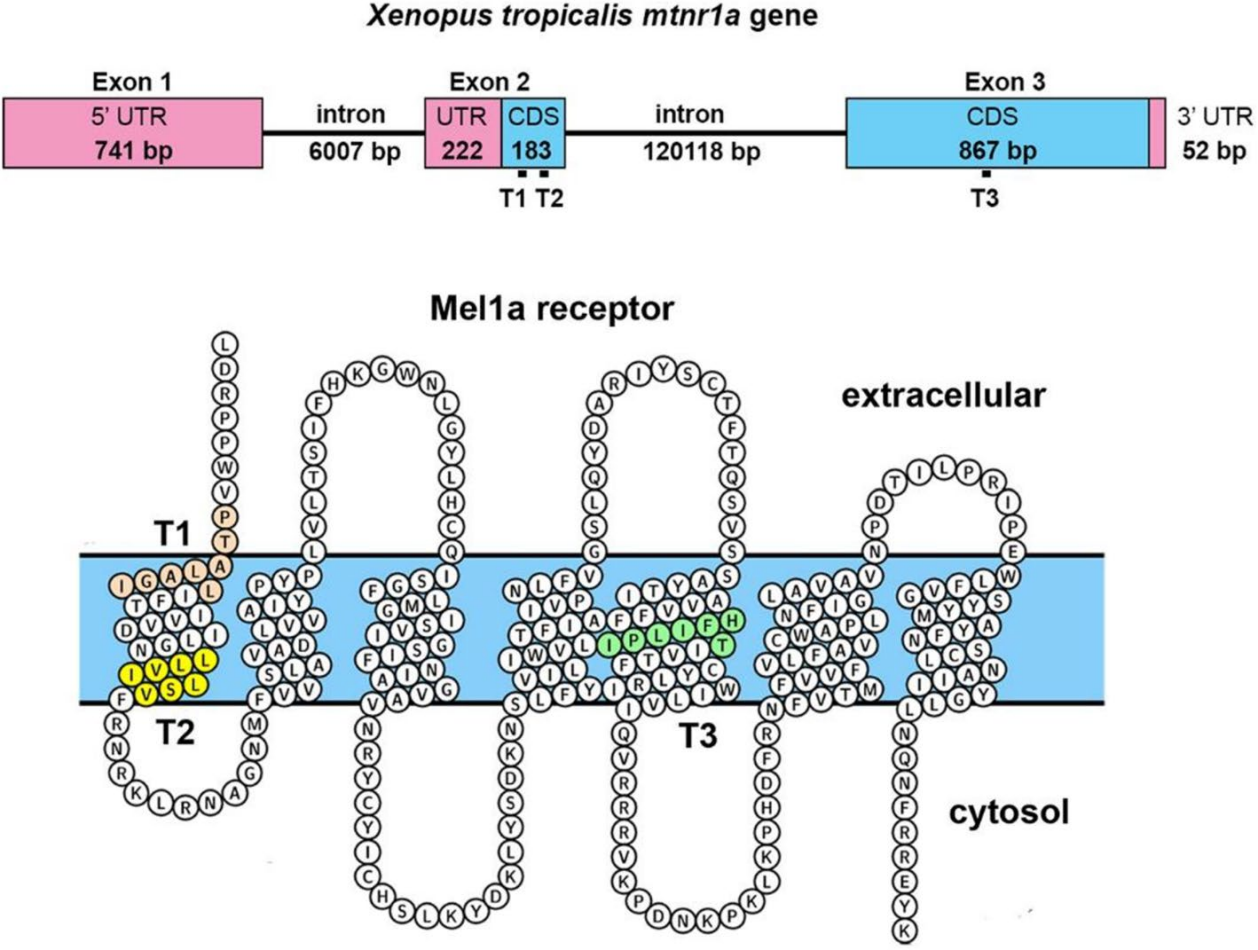
CRISPR/Cas9 sgRNA targeting of *X. tropicalis mtnr1a* gene. Upper image: Location of sgRNA targets T1, T2, & T3 are indicated by small black bars below the coding sequences (CDS) of exons 2 and 3 (blue boxes). The number of nucleotide base pairs (bp) is indicated in the exons and intervening introns. The 5′ and 3′ untranslated (UTR) regions are indicated by the red boxes. Lower image: Predicted conformation of *X. tropicalis* Mel1a melatonin receptor on the cell membrane (blue). The sites of the three sgRNA targets are indicated by colored residues in the first and fifth transmembrane domains. The receptor diagram (with the N- and C-terminal ends omitted for space) was created on the GPCRdb.org website (https://gpcrdb.org) ^74^.

We examined the impacts of disrupted melatonin receptor signaling on retinal cell function and viability, by mutating *mtnr1a* receptor gene expression of the Western clawed frog *Xenopus (Silurana) tropicalis*. We used in vivo CRISPR/Cas9 methodologies^28^ to create F0 founders, and subsequently generated F1 *X. tropicalis* progeny that are heterozygous for the expression of the Mel1a melatonin receptor with a predicted three-amino acid (VIL, residues 48–50) in-frame deletion in the first transmembrane domain (Fig. 1). We report here that heterozygous *mtnr1a* VIL deletion mutant tadpoles exhibit pervasive rod photoreceptor dystrophy and/or degeneration, whereas cone photoreceptors are relatively more abundant. The F2 homozygous mutant progeny of F1 *mtnr1a* VIL heterozygous mutants did not exhibit the same level of rod decline that was observed in retinas of their heterozygous mutant siblings. The observation that a small in-frame melatonin receptor mutation causes aggressive rod photoreceptor degeneration in heterozygous *X. tropicalis* supports the concept that circadian signaling is necessary for photoreceptor homeostasis and development.

## Results

### CRISPR sgRNAs direct Cas9 cDNA cleavage in the *mtnr1a* exon 2 coding region

We created mosaic founder mutant (F0 crispant) *X. tropicalis* animals by microinjecting one-cell embryos with Cas9 protein complexed with single-guide (sg) RNAs targeting coding regions in exon 2 (T1 and T2 sgRNA) and/or exon 3 (T3 sgRNA) of the *mtnr1a* gene (Fig. 1 and Supplementary Figs. S1-S3). The sgRNAs were injected singly or in two different combinations of each other (i.e., T1 & T2, or T1, T2, & T3) with the initial intent of optimizing the potential for knockdown of Mel1a receptor expression. The T1 and T2 sgRNAs targeted the proximal and distal margins of the first transmembrane domain of the Mel1a receptor protein, respectively, and the T3 sgRNA targeted the middle of transmembrane domain 5 (Fig. 1 and Supplementary Figs. S1-S3).

Blitz et al*.*^29^ demonstrated that up to two mismatches in the *Xenopus* sgRNA are tolerated well by the Cas9 enzyme, resulting in zero off-target mutations (of 619 sites analyzed). We accepted only one or zero mismatches in our sgRNA design, which provides greater assurance that our sgRNAs are unlikely to cause undesirable off-target effects. Furthermore, a BLAST search of the *X. tropicalis* genome database (Nigerian version 9.1) on Xenbase (https://www.xenbase.org/entry/) revealed no off-target sites for any of our three sgRNAs used in this study.

sgRNAs targeting Cas9 endonuclease cleavage of the DNA coding region of exons 2 and 3 of the Mel1a gene *(mtnr1a)* were synthesized in vitro and screened for their ability to guide Cas9 protein to the DNA target site (Supplementary Fig. S1). Incubation of *mtnr1a* PCR-amplified DNA templates with Cas9 protein and each of three different sgRNAs showed that each sgRNA was effective in directing targeted Cas9 cleavage of DNA (Supplementary Fig. S2). The T2 sgRNA, which targets the cytoplasmic end of the transmembrane domain 1 in exon 2, was more effective (83%) than the T1 sgRNA (69%) and T3 sgRNA (43%) (Supplementary Fig. S2). The relative values were consistent with the projected targeting efficiencies analyzed by CHOPCHOP (https://chopchop.cbu.uib.no/). The online tool inDelphi identifies sgRNA sequences with predictable editing outcomes (https://indelphi.giffordlab.mit.edu/) ^30^ to show that the T2 sgRNA in this study is 27% likely to cause a 9-bp deletion and 18% likely to cause a 12-bp deletion. We also used CRISPRdirect (https://crispr.dbcls.jp/) analysis of the 20-mer T2 sgRNA in this study to show that *no* off-targets occurred in the coding exons elsewhere in the *X. tropicalis* genome^31^.

### F0 crispant frogs express a variety of indel mutations targeted by the *mtnr1a* T2 sgRNA

PCR products amplified from genomic DNA (gDNA) extracted from tail clips of age- and stage-matched wild type (WT) or F0 tadpoles were analyzed by T7 endonuclease I (T7E1) mismatch cleavage assays^32^ and direct Sanger DNA sequencing. F0 tadpoles were initially screened by T7E1 assays in which mismatched (i.e., heterozygous WT and mutant alleles) re-annealed PCR products amplified from gDNA templates are cleaved at the site of the mismatch by the T7E1 enzyme, generating two distinct PCR fragments (Supplementary Fig. S4). Direct sequencing of PCR products from F0 *mtnr1a* mutant gDNA revealed degradation of chromatogram peaks at or just downstream of the T2 PAM target site, such as the VIL cleavage site located 4-bp downstream of the PAM site (Supplementary Fig. S4). In contrast to the T2 sgRNA, we detected only a few mutants resulting from the T1 sgRNA, and no mutants from the T3 sgRNA. Thus, mutations of the T1 and T3 regions of the *mtnr1a* receptor gene were not pursued for further study.

Deconvolution of DNA sequences by TIDE analysis (tracking of indels by decomposition; https://tide.nki.nl) ^33^ reveals the difference in aberrant indel sequences between WT and mutant animal sequences. TIDE analysis illustrates the range of indel sizes and frequency of expression in four individual F0 founder animals (Supplementary Fig. S5). The T2 sgRNA targeting efficiency varied from 44.6 to 86.2% among the four F0 crispants analyzed, and all animals displayed both insertions and deletions near the PAM T2 site. Notably, a 9-bp non-frameshift deletion was detected in three of the four crispants, which was the size of the prominent deletion mutation that we later observed in some F1 heterozygotes, including animals with the 9-bp VIL deletion that causes photoreceptor degeneration. WT and VIL homozygote samples showed no significant allelic variance compared to the reference trace, as expected.

Genomic DNA was extracted from web clips of five mutant F0 sibling founder frogs raised to sexual maturity. The *mtnr1a* target areas were PCR-amplified and cloned into plasmids for DNA sequencing to identify specific indels represented in the mosaic mixture of mutations. Many clones (59 total) displayed mutations in the T2 target area of exon 2 (Supplementary Fig. S6), which corresponds to the cytoplasmic end of the first transmembrane domain of the Mel1a receptor (Fig. 1, Supplementary Figs. S1, S3). Of the 59 mutant sequenced clones, there were 23 different indel or substitution mutations identified in the T2 target area (Supplementary Fig. S6).

### The VIL deletion mutation of the *mtnr1a* receptor gene causes rod photoreceptor degeneration in heterozygous tadpoles

**Figure 2:**
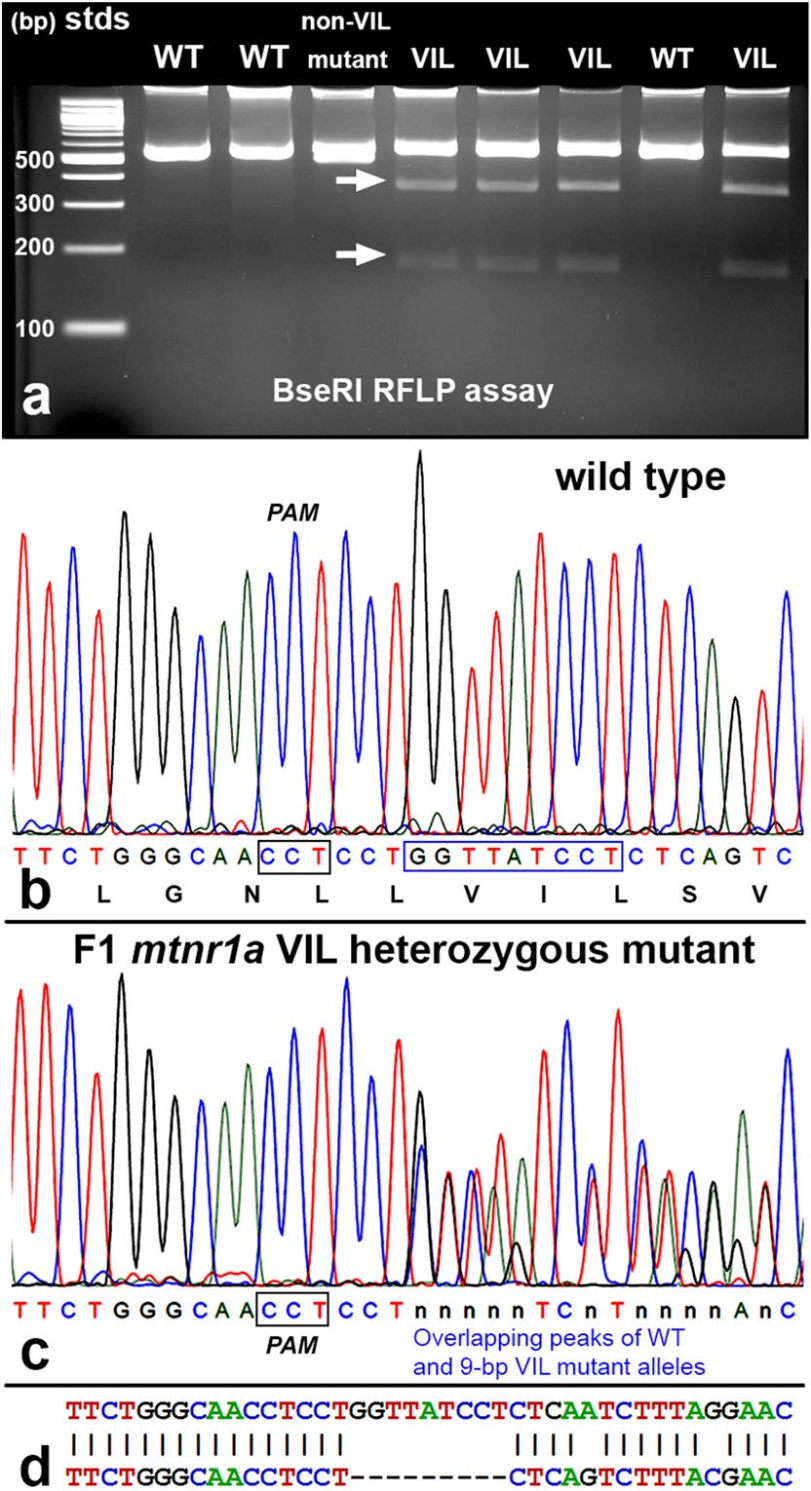
Identification of heterozygous F1 animals with CRISPR/Cas9 indel mutations of the *mtnr1a* gene. (**a**) Representative RFLP analysis shows that PCR products of *mtnr1a* VIL heterozygotes display two predicted cleavage bands (arrows), whereas WT and non-VIL mutant DNA is not cleaved. The creation of a BseRI site by the VIL deletion is illustrated in Supplementary Fig. S3. (**b**) Direct PCR Sanger sequencing chromatogram of F1 WT progeny display prominent peaks for all bases in the T2 sgRNA target area. The T2 PAM sequence (CCT) is boxed in black, and the downstream 9-bp sequence of the *mtnr1a* VIL deletion mutation is boxed in blue. The corresponding amino acid sequence is shown below the nucleotide codon sequence. (**c**) In an F1 tadpole heterozygous for the *mtnr1a* 9-bp (VIL) deletion, the trace decomposes (i.e., mixed base calls) at the predicted target site 4-bp downstream of the T2 PAM site, and continues to the end of the trace, as expected for a heterozygous indel mutation. The decomposed sequence represents the overlapping peaks of the *mtnr1a* WT and VIL alleles. (**d**) Partial view of alignments from Poly Peak Parser (https://yosttools.genetics.utah.edu/PolyPeakParser/) output revealing the heterozygous 9-bp *mtnr1a* VIL deletion mutation. The WT allele is the top line and the mutant allele is the bottom line.

When the F0 founders reached sexual maturity, we outcrossed them with WT *X. tropicalis* to assess which mutations were passed into the germline. T7E1 assays performed on PCR cDNA amplified from gDNA of F1 animals with different *mtnr1a* mutations revealed slightly different patterns of *mtnr1a* cleavage products (Supplementary Fig. S7), consistent with inheritance from mosaic F0 founders. T7E1 assays reliably identified mutant F1 tadpoles from WT, but did not identify the specific mutations expressed. Phenotyping and genotyping of F1 tadpoles revealed a correlation of ROS loss with a 9-bp (GGTTATCCT) deletion mutation starting 4-bp downstream of the T2 PAM site at the end of exon 2 of the *mtnr1a* gene (Supplementary Fig. S8). The 9-bp deletion was predicted to cause an in-frame 3-amino acid (valine-isoleucine-leucine; VIL) deletion. F1 tadpoles were definitively identified as *mtnr1a* VIL deletion mutants by direct PCR sequencing, sequencing of plasmid clones, and restriction fragment length polymorphism (RFLP) analyses. The VIL deletion created a unique BseRI endonuclease restriction site (CTCCTCT; Supplementary Fig. S8), rendering it useful for RFLP screening of F1 tadpoles. Incubation of PCR products of gDNA from VIL *mtnr1a* mutants with BseRI endonuclease cleaved the 530-bp PCR product into 189-bp and 341-bp fragments, as predicted (Fig. 2a). This RFLP assay positively identified 100% of the F1 heterozygous *mtnr1a* VIL mutants, as confirmed by sequencing of PCR products (Fig. 2b,c) and plasmid clones (Supplementary Fig. S9). DNA sequence chromatograms of plasmid clones of F1 *mtnr1a* VIL mutant animals show no sequence degradation, but instead have 9-bp deletions (GGTTATCCT) beginning at the identical 4-bp downstream site (Fig. 2c,d). The results of these analyses confirmed the prediction by inDelphi^30^ of a high rate of expression of the 9-bp deletion mutation.

**Figure 3:**
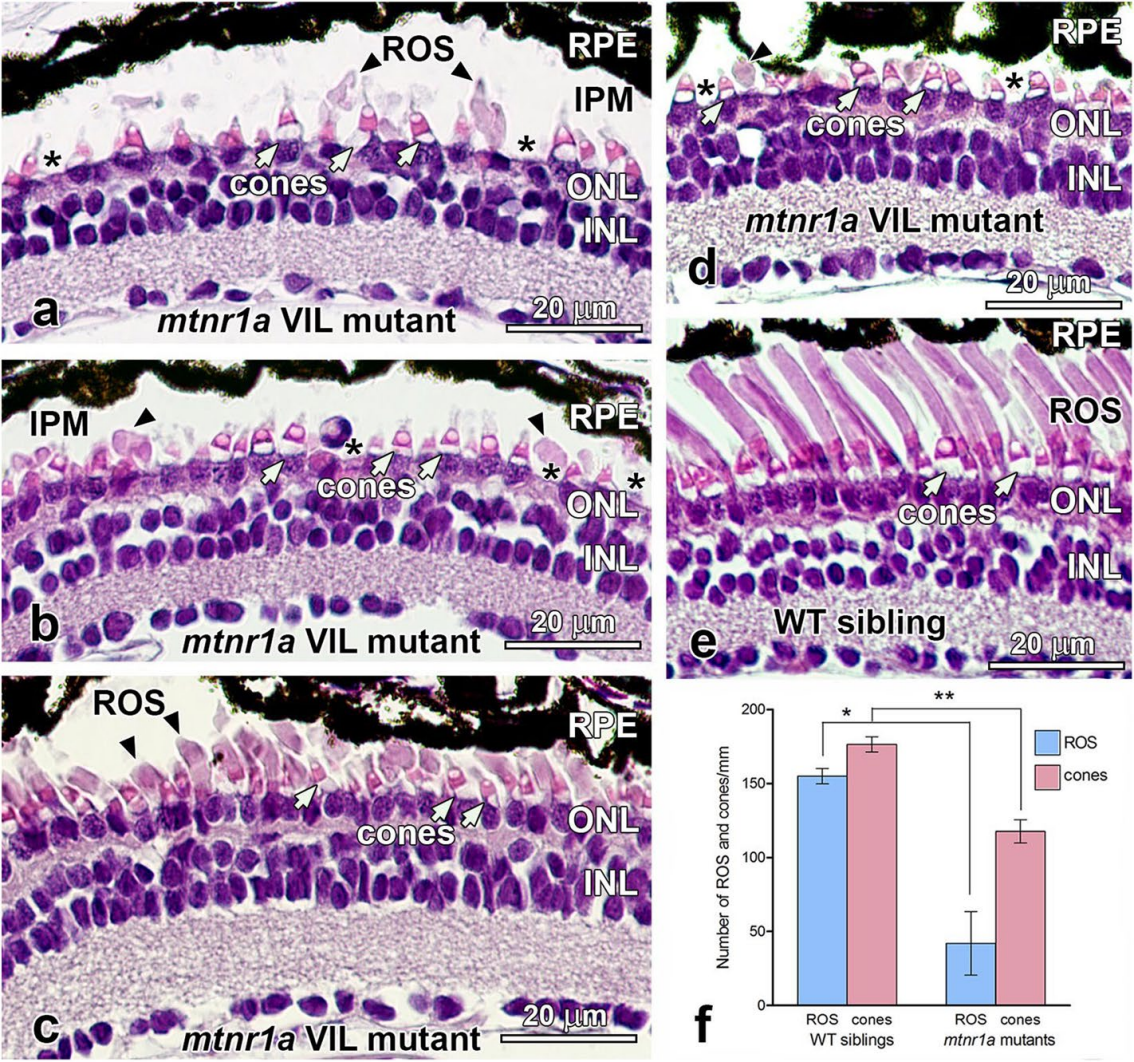
Photoreceptors degenerate in stage 50–52 (35-day-old) heterozygous *mtnr1a* VIL mutant tadpoles. (**a**–**d**) H&E-stained paraffin sections of the central retina of four different stage 50–52 *mtnr1a* VIL mutant tadpoles display rod loss and/or dystrophy. Retinas of mutant tadpoles have normal appearing cones (white arrows), but severely dystrophic rod outer segments (ROS; black arrowheads). Note the frequent absence of rod inner segments (asterisks). (**b**) A presumptive leukocyte is in the area of the interphotoreceptor matrix (IPM) at the center of the panel. (**c**,**d**) Some ROS persist in the mutant central retinas, but are much shorter than the ROS of WT sibling retinas (**e**), and exhibit some dystrophy. (**e**) Retinas of WT siblings exhibit healthy rod and cone photoreceptors. (**f**) Quantitative analysis of ROS and cones in WT vs VIL central retinas. The average number of ROS (blue bars) or cones (red bars) in 6–12 different retinal sections from four different animals (**a**–**d**) in each group (i.e., n = 4) were analyzed for statistical significance by an unpaired two-tailed *t*-test. Error bars indicate standard error of the mean (SEM). In mutant retinas, the number of ROS declined by 73% (*; *p* = 0.0022), and cones declined by 33% (**; *p* = 0.0008) as compared to WT. *RPE* retinal pigment epithelium, *ROS* rod outer segments, *ONL* outer nuclear layer, *INL* inner nuclear layer, *IPM* interphotoreceptor matrix, cone inner segments; white arrows, ROS; black arrowheads, inner segment layer; asterisks. Magnification bars = 20 µm.

F1 heterozygous progeny of F0 founder *mtnr1a* CRISPR/Cas9-edited frogs were analyzed morphologically at various stages of development by routine H&E histology. Of the over 200 *mtnr1a* F1 tadpoles or froglets examined, obvious and consistent rod degeneration was present in only the 9-bp VIL deletion mutants. The absolute number of F1 VIL mutants reported here do not necessarily represent the relative frequency of mutation expression, since our analyses were biased to identify animals with mutations detected by T7E1 assays that were then randomly confirmed by sequencing of plasmid clones. Of the 22 mutant animals in which the *mtnr1a* region was cloned and sequenced, 54.5% (12/22) exhibited a VIL deletion (Supplementary Fig. S9). Histologic examination of 35-day-old F1 pre-metamorphic VIL mutant tadpoles (stages 50–52) revealed massive loss of rod photoreceptors (Fig. 3). The rod degeneration was most pronounced in the central region (near the optic nerve head). The severity of photoreceptor degeneration displayed some individual variation, insofar as retinas of some animals were almost devoid of rod cells (Fig. 3a,b,d), whereas others had a substantial number of rods, many of which had truncated or otherwise dystrophic outer segments (Fig. 3c).

The number of rod photoreceptors, as determined by number of ROS per mm in the central retina, was reduced by 73% (unpaired two-tailed *t*-test; *p* = 0.0022) at stages 50–52 (35-day) in *mtnr1a* VIL mutant tadpoles, compared to age- and stage-matched WT siblings (Fig. 3f). In comparison, cone photoreceptors were less affected, although the average number of cones per mm declined by 33% (*p* = 0.0008) in the VIL mutant tadpoles (Fig. 3f). The relative difference in means (cells/mm) between WT and mutants was 113.00 ± 22.12 (standard error of the mean; SEM) for ROS, and 58.75 ± 7.38 for cones. Notably, cell nuclei of unknown origin were often observed in the outer plexiform layer (OPL), which is where photoreceptor terminals contact retinal interneurons, and were especially prominent in areas of severe rod loss (Fig. 3a,b,d). There were no obvious morphologic aberrations in the RPE in the 35-day heterozygous *mtnr1a* VIL mutant retinas. We did not detect morphologic deficits in retinas of tadpoles at earlier developmental stages (about stages 46–48; 9-and 16-days old).

**Figure 4:**
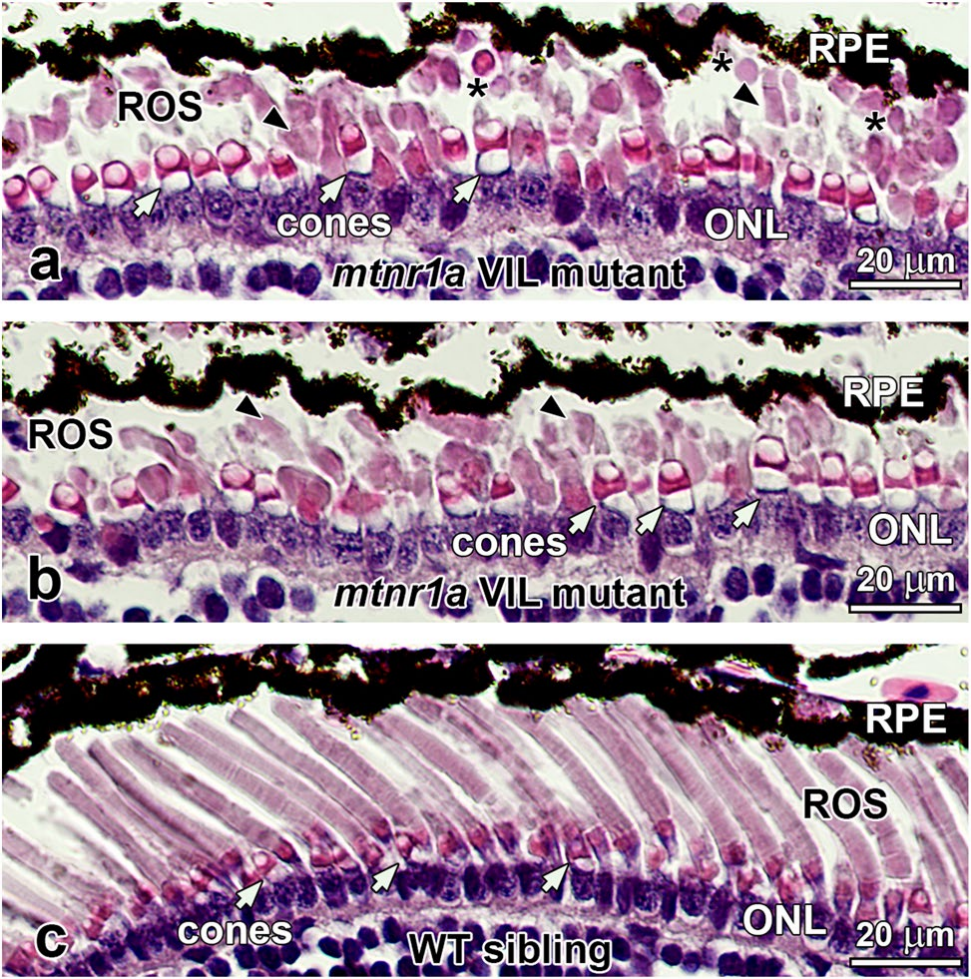
Rod photoreceptors are dystrophic in stage 54–56 (48-day-old) heterozygous *mtnr1a* VIL mutant tadpoles. (**a**,**b**) H&E-stained paraffin sections of central retina from a 48-day VIL mutant tadpole displays ROS dystrophy (black arrowheads), whereas cones appeared relatively normal (white arrows). Single or clusters of large shed ROS (asterisks) were often present in the interphotoreceptor matrix. (**c**) Retinas of WT siblings exhibit healthy-appearing rod and cone photoreceptors. *RPE* retinal pigment epithelium, *ROS* rod outer segments, *ONL* outer nuclear layer, *INL* inner nuclear layer, cone inner segments; white arrows, ROS; black arrowheads, shed ROS tips; asterisks. Magnification bars = 20 µm.

Tadpoles at stages 54–56 (48-day) displayed central rod dystrophy and degeneration (Fig. 4). Cones were abundant and appeared to have normal morphology (Fig. 4a,b). Clusters of shed ROS tips that had not been phagocytized by the RPE were often seen in the interphotoreceptor matrix (IPM; Fig. 4a). The presence of large, un-phagocytized shed ROS tips in the IPM is abnormal, since ROS shedding and RPE phagocytosis is synchronized to occur at about 2 h after light onset. Serial sections revealed that many of the ROS profiles were indeed shed ROS packets rather than obliquely-oriented intact ROS. Tissues of the 48-day tadpoles were fixed at nine hours after lights on (i.e., late afternoon), which is well beyond the time where shed large ROS tips are typically observed in the normal retina^34^. Rod degeneration in the stage 54–56 (48-day) F1 pre-metamorphic VIL mutant tadpoles was more severe in the central region of the retina than in the dorsal or ventral regions of the peripheral retina (Supplementary Fig. S10). The average ROS length in the central retina of *mtnr1a* VIL mutants decreased by 64%, compared to WT siblings. Rod degeneration was consistently less severe in the peripheral regions, with the ventral region somewhat less affected than the dorsal region (Supplementary Fig. S10).

### Homozygous *mtnr1a* VIL tadpoles do not exhibit obvious rod degeneration

We raised to adulthood a homozygous F1 *mtnr1a* VIL male frog that resulted from the crossing of two mosaic F0 founders that expressed the VIL deletion in their germline (i.e., crispants #1 and #3 in Supplementary Figs. S4-S6). We crossed this F1 mutant male to a heterozygous VIL female sibling, which produced an F2 generation with a 50/50 ratio of homo-/heterozygous VIL mutant progeny. We also crossed other mature F1 heterozygous VIL mutant siblings with each other to generate F2 heterozygous and homozygous progeny. We genotyped these animals with T7E1 assays in tandem with BseRI RFLP assays and DNA sequencing. Together, these genotyping assays definitively identified the F1 and F2 progeny as WT or VIL homozygous or heterozygous mutants (Supplementary Fig. S11).

**Figure 5:**
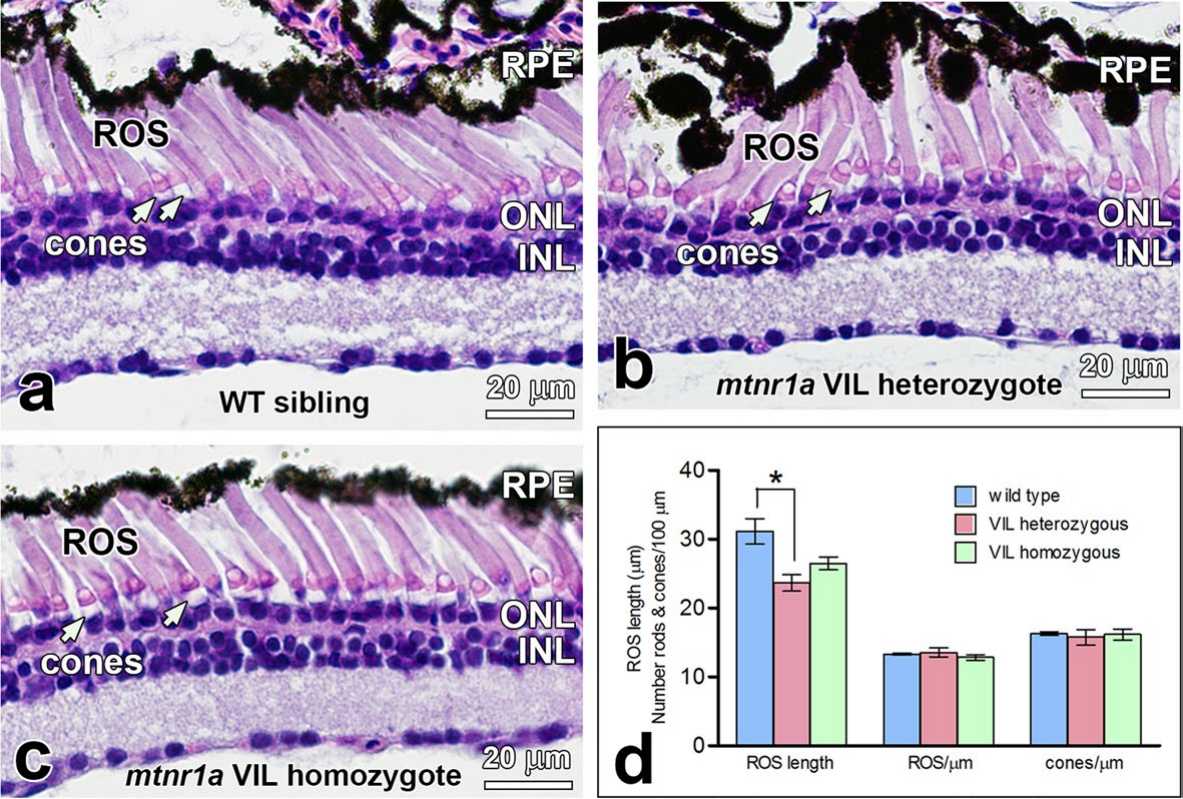
Rods are dystrophic in stage 54–56 (4-month-old) F2 heterozygous but not homozygous *mtnr1a* VIL mutant tadpoles (n = 5). F2 heterozygous and homozygous tadpoles were generated by crossing two F1 VIL mutant heterozygotes. (**a**) WT siblings of F2 mutants exhibit healthy-appearing rod and cone photoreceptors. (**b**) Central retinas of VIL mutant heterozygous tadpoles display ROS dystrophy and abnormal distribution of RPE cells, whereas cones appear relatively normal (white arrows). (**c**) Retinas of homozygous VIL mutant siblings display morphologies similar to WT siblings. (**d**) Quantitative measurements of rod dystrophy in *X. tropicalis* retinas show that rod outer segment (ROS) length is diminished significantly by 24% in VIL heterozygous 4-month-old tadpoles, compared to WT when analyzed by an unpaired two-tailed *t*-test (*; *p* = 0.0267, n = 3). The 15% decline in ROS length in VIL homozygous tadpoles compared to WT was statistically insignificant (*p* = 0.0867, n = 3). No significant differences in rod (i.e., ROS) or cone abundance were observed among the three genotype groups. Measurements were made of digital images of groups of 4-month-old tadpoles represented in **a**–**c**. Error bars indicate standard error of the mean. *RPE* retinal pigment epithelium, *ROS* rod outer segments, *ONL* outer nuclear layer, *INL* inner nuclear layer, cone inner segments; white arrows. Magnification bars = 20 µm.

Phenotype analysis revealed no obvious retinal degeneration in F2 *mtnr1a* VIL homozygotes (Fig. 5). We analyzed F2 *mtnr1a* VIL mutant heterozygous and homozygous sibling tadpole phenotypes (n = 5) at 4 months of age (stage 54–56), which is a somewhat advanced age for this stage of tadpole. In our facility, tadpoles typically undergo metamorphosis between 2 and 6 months of age (metamorphosis at about 2 months of age is considered optimal in most laboratory settings), possibly due to subtle differences in population density or food intake. Thus, animals undergoing metamorphosis ranged in age from 2 to 4 months, which is why all control animals in this study were both age- and stage-matched with their mutant counterparts. In these VIL heterozygotes, we observed some moderate ROS dystrophy (Fig. 5b), but not as massive as seen at 35 and 48 days (Figs. 3 and 4). In contrast, WT siblings (Fig. 5a) and *mtnr1a* VIL homozygous siblings (Fig. 5c) appeared to have relatively normal morphology.

The number of rods and cones appeared to be the same in the WT, VIL heterozygous, and VIL homozygous 4-month-old tadpole retina sections of tadpoles represented in Fig. 5a–d. However, average ROS length was significantly lower (24%) in VIL heterozygous animals compared to WT, as measured by an unpaired two-tailed *t*-test (*p* = 0.0267). The ROS length of VIL homozygous animals were on average 15% lower than WT, but were not statistically different from WT siblings (*p* = 0.0867). These data provide quantitative support that VIL heterozygotes exhibit a moderately more severe degenerative phenotype than do the VIL homozygous mutant tadpoles examined in this study. Because of the inherent individual and developmental variation of phenotype severity, further analysis of additional animals could enable more detailed analyses of the mutant phenotypes.

### Mel1a receptor proteins are expressed at WT levels in VIL heterozygote and homozygote retinas

**Figure 6:**
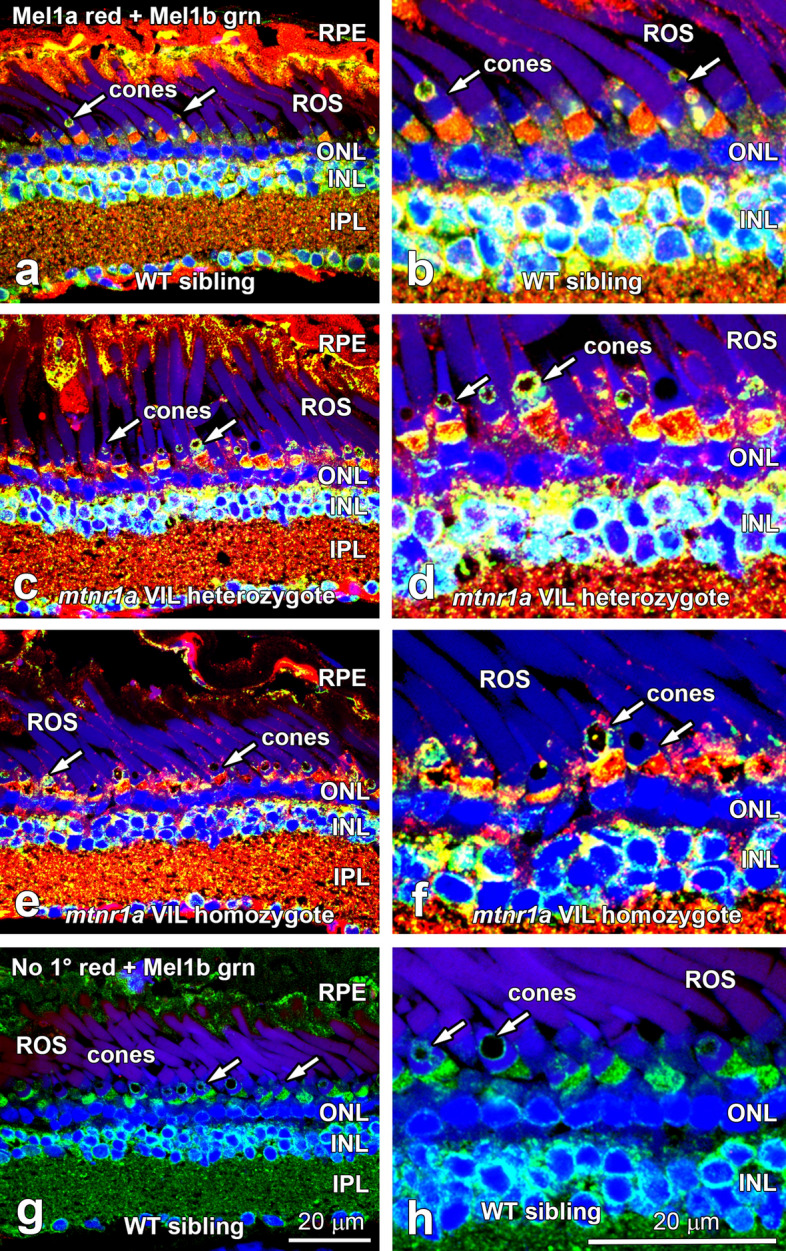
Retinas of heterozygous and homozygous *mtnr1a* VIL mutant tadpoles display Mel1a protein expression at intensities similar to WT siblings. Double-label confocal immunohistochemistry (IHC) of (**a**,**b**) WT, (**c**,**d**) F2 VIL heterozygous, and (**e**,**f**) F2 VIL homozygous tadpole head paraffin sections was performed with antibodies to *X. laevis* Mel1a (red label) and Mel1b (green label) melatonin receptors. Panels on the right are higher magnifications of areas in panels on the left side of the figure. Mel1a receptor fluorescent signal intensity and location appears to be similar in animals of all three genotypes. The Mel1a receptor is expressed in RPE, rod and cone photoreceptors (arrows), and inner retinal neurons. The Mel1a and Mel1b receptors display an expression pattern different from each other, although there are areas in which the red and green labels merge as a yellow label, suggestive of co-expression and/or very close proximity to each other. (**g**,**h**) WT retina sections processed for IHC with Mel1b antibodies, but without Mel1a antibodies (No 1° red) display specific immunofluorescence for Mel1b (green), but not for Mel1a (red). *RPE* retinal pigment epithelium, *ROS* rod outer segments, *ONL* outer nuclear layer, *INL* inner nuclear layer, *IPL* inner plexiform layer, arrows; rod photoreceptor inner segments. Magnification bars = 20 µm.

Immunohistochemistry (IHC) was used to determine if VIL mutant Mel1a receptor proteins of VIL heterozygous and homozygous animals are expressed at comparable levels as WT receptor proteins in the retina and other tissues. The specificities of the antibodies to *X. laevis* melatonin receptors have been previously reported^35–38^. Retinas of tadpoles in all three genotype groups displayed about the same intensity and localization of expression of the Mel1a receptor protein (Fig. 6a–f). It is important to note that since immunofluorescence is not considered to be a quantitative measure, no attempt was made to quantify differences in immunolabeling intensities. Particularly, Mel1a receptor immunoreactivity was prevalent on or near the plasma membrane of the photoreceptors, suggesting that some of the receptor is trafficked to the cell surface. Mel1a immunoreactivity was especially robust in cone inner segments in all animals tested. Retina sections incubated without primary antibody displayed no specific immunoreactivity (Fig. 6g,h).

### Post-metamorphic heterozygous *mtnr1a* VIL deletion mutant adult frogs display abundant rod photoreceptors

**Figure 7:**
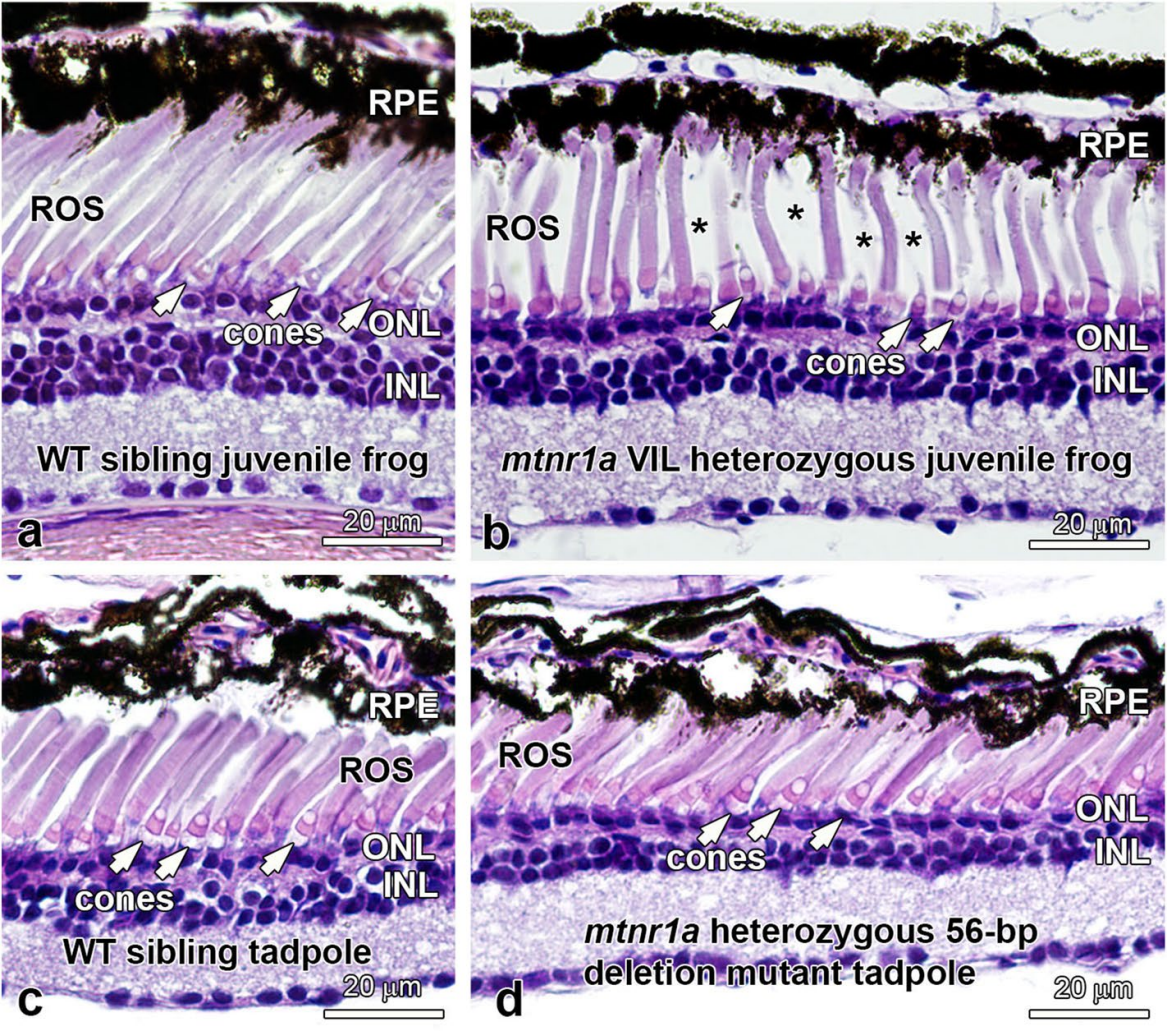
Heterozygous *mtnr1a* VIL mutant young adult (juvenile) frogs display normal rods and cones. (**a**) WT juvenile frogs display normal rods (ROS) and cones. (**b**) F1 *mtnr1a* VIL heterozygous mutant juvenile sibling frogs display healthy-looking rods and cones, although the ROS density appears to be lower than in WT (asterisks). Mtnr1a 56-bp deletion mutant tadpoles do not display rod dystrophy. (**c**) Four-month-old WT tadpole retinas and (**d**) F1 *mtnr1a* heterozygous 56-bp deletion (potential knockout) siblings exhibit healthy-appearing rods (ROS) and cones (white arrows). *RPE* retinal pigment epithelium, *ROS* rod outer segments, *ONL* outer nuclear layer, *INL* inner nuclear layer. Magnification bars = 20 µm.

In contrast to the obvious rod degeneration observed in mutant tadpoles, young adult post-metamorphic (stage 66; 12-month) *mtnr1a* VIL heterozygous mutant frogs displayed abundant ROS, although their density was slightly lower (about 15%) in mutant retinas compared to those of WT siblings (Fig. 7a,b). Only two young adult VIL homozygous frogs were available for morphologic analysis, since all other homozygous animals were used for the earlier developmental analyses. In these two animals, we did observe significant ROS dystrophy, but the n = 2 limits our confidence in these observations, which need to be addressed further when the next generation of tadpoles are raised to sexual maturity.

### Large frameshift deletion mutation of the *mtnr1a* receptor does not result in rod degeneration

**Figure 8:**
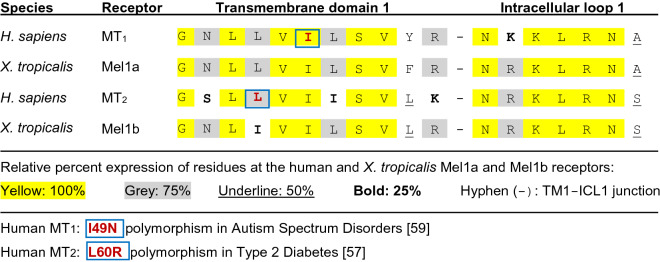
Amino acid alignment of the endofacial portion of the first transmembrane domain of human and *X. tropicalis* melatonin receptors. The hydrophobic residues of transmembrane domain 1 (TM-1) located near the membrane cytoplasmic surface is highly conserved among GPCRs, as exemplified by the alignment of this region in the human MT1 and MT2 receptors and the homologous *X. tropicalis* Mel1a and Mel1b receptors. The human MT1 and *Xenopus* Mel1a receptors show a 100% amino acid identity in the GNLLVILSV 9-bp region in TM-1. The first intracellular loop (ICL-1) is also highly conserved, as indicated in yellow. The *mtnr1a* T2 sgRNA most effectively targeted the hydrophobic LLVIL residues (see Supplementary Fig. S6) in the center of the *Xenopus* Mel1a endofacial TM-1 region illustrated here. This region is the site of a human MT1 nonsynonymous point mutation (I49N) associated with autism^59^ and a human MT2 mutation (L60R) linked to type 2 diabetes^57^. The amino acid point mutations associated with each disorder are indicated by a blue box.

To examine the potential for *mtnr1a* knockout mutations, we analyzed F1 heterozygous tadpoles that express a 56-bp deletion mutation predicted to knock down Mel1a protein expression. The 56-bp deletion includes the exon 2/intron 2 splice site, so deletion of the splice site may potentially allow translation to continue into the intron (Supplementary Fig. S12). No premature stop codons appeared in the 270-bp of readable sequence downstream from the end of the deletion. The potential that such an aberrant protein sequence would be translated in the mutant cells is improbable, because the mRNA would potentially enter the nonsense-mediated decay pathway and/or the mutant receptor protein would likely be degraded and thus not trafficked to the cell surface. It is therefore likely that this mutation causes knockdown of receptor expression and/or trafficking, but it requires further study. We did not observe any obvious deleterious impacts on rod survival in these animals (Fig. 8c,d), and we thus tentatively suggest that tadpoles that are heterozygous for the *mtnr1a* 56-bp deletion mutation do not display the severe degenerative phenotype as seen in the heterozygous VIL deletion tadpoles.

## Discussion

To examine the role of melatonin receptor signaling on retinal circadian functions, we mutated the *mtnr1a* melatonin receptor gene of *X. tropicalis* using CRISPR/Cas9 and generated heterozygous F1 progeny, and analyzed their retinal morphology. Unexpectedly, we observed a massive loss of rod photoreceptors in F1 heterozygous mutant tadpoles with a 9-bp deletion near the end of exon 2 of the Mel1a receptor (Figs. 3,4). This mutation is predicted to create a three amino acid (valine-isoleucine-leucine; VIL) non-frameshift deletion near the cytosolic end of the first transmembrane (TM-1) domain of the Mel1a receptor protein (Supplementary Figs. S1, S3). This phenotype was surprising, since earlier studies using MT1 and MT2 knockout mice revealed rather subtle temporal shifts in some circadian functions of the retina^39,40^ and a moderate reduction in cone viability in aged knockout mice^41^. We observed rod degeneration in *mtnr1a* VIL heterozygous mutant tadpoles as early as day 35 post-hatching (about stages 50–52). There was some individual variation in severity of rod degeneration at all stages examined (Fig. 4), which is typical of inherited heterozygous mutations^42,43^. Until the phenotype of homozygous adult frogs is further documented, we suggest cautious interpretation of the potential events that cause the dystrophic changes that occur in rods of developing tadpoles.

Dimerization of the Mel1a VIL mutant with WT Mel1a, 1b, or 1c receptor protomers in the endoplasmic reticulum (ER) may divert the VIL/WT dimer into the proteasome degradative pathway, causing a dominant loss-of-function. Additionally, retention of mutant melatonin receptors in the ER may be detrimental to photoreceptor health due to ER stress^44–47^. Also, VIL/WT dimers may impede normal WT homodimer signaling by forming complexes that are uncoupled from the internalization and sequestration pathways that are crucial for normal down-regulation of receptors. This could keep them active during periods of chronic exposure to radiant light energy, which could cause a loss-of-function of the WT receptor subtypes. We observed that Mel1a immunoreactivity is present on or near the cell surface of photoreceptors and other retinal cells of WT and VIL mutants (Fig. 6). Thus, some Mel1a VIL/WT dimers could feasibly be trafficked to the plasma membrane and assume an aberrant role in melatonin signaling.

In a rod gain-of-function model, constitutive activation (i.e., during both night and day) of the receptor could increase susceptibility of the rods to light-induced photoreceptor degeneration. This hypothesis is supported by evidence that treatment with melatonin during periods of light exposure is detrimental to long-term rod photoreceptor viability^8^. Dimerization of GPCR monomers (including MT1/MT2) is widely-considered to be essential to receptor functions^48^. Fukami et al.^48,49^ have proposed a paradoxical gain-of-function mechanism in which most WT GPCRs reside on the cell surface as homodimers or heterodimers under basal conditions, and that the presence of mutant/WT receptor dimers on the cell surface can negatively affect the activity and/or cellular regulation of the WT dimer partners. The authors described a ‘paradoxical’ gain-of-function of the GPCR prokineticin receptor 2 (PROKR2)^49^. In this example, *PROKR2* mutation in a girl with precocious puberty *enhanced* signal transduction in vitro when *co-expressed with the WT receptor*, compared to expression of the mutant (i.e., homozygous ) or WT receptors alone. PROKR2 is known to exist as a dimer in vivo^50^.

One of the earliest reports of an activating mutation in the TM-1 helical domain was of the lutropin receptor^51^. The TM-1 domain is also the general location of the Mel1a receptor VIL deletion in *Xenopus* in the current report. An alanine to valine substitution in TM-1 identified in a heterozygous lutropin receptor mutant human patient causes a 7.5-fold increase in cyclic AMP synthesis in vitro^51^. The authors suggest that structural changes induced by this mutation are likely to create significant conformational modifications, such as retaining the receptor in an activated state. Another pertinent example to support the precedent for GPCR dimerization via a TM-1 interface is the thromboxane A_2_ prostanoid receptor (TP)^52^. Dimerization (*i.e.,* FRET signal) and consequent increased signaling was evident in cell cultures co-expressing the two WT monomers. However, receptor dimerization did *not* occur in cells expressing only a TM-1 mutant pair (representative of a *homozygous* mutant genotype), or a WT and TM-1 substitution mutant TP monomer (i.e.,* heterozygous* equivalent). This study provided evidence that hydrophobic residues of GPCR TM-1 domains may be necessary for dimer formation of some GPCRs. Further, ligand-promoted internalization of WT TP receptor occurred only when both α and β isoforms (which form heterodimers in vivo) were co-expressed^52^. Another study has shown that ligand-induced inositol phosphate signaling is enhanced in vitro in cells that co-express both α and β monomers, compared to either protomer individually^53^.

In an RPE or rod loss-of-function model, the mutant Mel1a receptor protein dimerizes with WT Mel1a receptor in the ER, which could divert both receptors into the proteasome degradative pathway rather than to the cell surface. This would severely reduce WT receptor expression at the cell surface, which is a well-known mechanism by which GPCR mutations exert dominant negative phenotypes^49^. In support of this scenario, loss of circadian Mel1a receptor signaling impairs the ability of mouse RPE cells to phagocytize shed rod outer segments at the appropriate circadian time^39^. Furthermore, deficits in circadian RPE functions potentially lead to photoreceptor degeneration in many species, including humans^54,55^. Rod degeneration, due to retention of mutant receptors in the ER, is also a common scenario in several blinding disorders of humans^44,45,47^.

Since many organ systems throughout the body express melatonin receptors, one scenario is that aberrant Mel1a receptor function in other affected target organs could potentially contribute systemically to the degenerative phenotype observed in the *mtnr1a* VIL retina. The high metabolic activity of photoreceptors makes them particularly vulnerable to metabolic disorders^56^, so they may manifest degenerative responses earlier than cells in other organ systems. Therefore, the concept that systemic disturbances in melatonin signaling might also contribute to the retinal phenotype should be considered and tested.

The region of the *X. tropicalis* Mel1a receptor that was most effectively targeted for CRISPR/Cas9 mutagenesis in this study was the cytoplasmic end of TM-1 (Fig. 1, Supplementary Figure S1). There is a high degree of amino acid homology in this hydrophobic region among the human MT1 and MT2 receptors and the *X. tropicalis* Mel1a and Mel1b receptors (Fig. 8). A nine-amino acid sequence (GNLLVILSV) in this region displays 100% identity between the human MT1 and *X. tropicalis* Mel1a receptors, and these same regions of the human MT2 and *X. tropicalis* Mel1b receptors display 89% (8/9) amino acid identity. Similarly, the first intracellular loops (ICL-1) show high degrees of homology between species and receptor subtypes (Fig. 8). The high homology of the human MT1 and *X. tropicalis* Mel1a receptors in this characteristically conserved region favors the functional relevance of mutational studies of the frog Mel1a receptor to human health.

In the RPE of *X. laevis*, Mel1a and Mel1c immunoreactivity is located on the apical microvilli, whereas Mel1b is localized primarily to the basolateral portion of the RPE membrane and cytoplasm^38^. This same pattern of melatonin receptor subtypes occurs in the *X. tropicalis* RPE (Fig. 6). Mel1a is localized to rod and cone photoreceptor inner segment plasma membrane and cytoplasm (Fig. 6), similar to the pattern seen in *X. laevis*. In *X. laevis* inner retina, Mel1a receptors are reported to be expressed in horizontal, amacrine, bipolar, and ganglion cells^35–38^. The pattern of Mel1a immunoreactivity in the *X. tropicalis* inner retina appears very similar to that reported earlier for *X. laevis*, but will require more in-depth analyses. The presence of Mel1a immunoreactivity in the retina of homozygous VIL mutants supports the concept that at least some of the mutant Mel1a protein is synthesized by retinal cells and trafficked to the cell surface.

A cluster of single-nucleotide polymorphisms (SNPs) of hydrophobic residues in TM-1 (*i.e.,* P36S, A42P, A52T, and L60R) of the human MT2 receptor is reported to be associated with type 2 diabetes (T2D)^57,58^. The L60R variant of the human MT2 receptor is located in the same highly conserved hydrophobic region of TM-1 as the *X. tropicalis mtnr1a* mutations described in the current study (Fig. 8). Moreover, genetic screening of a large European cohort revealed that in one autistic child, a heterozygous MT1 nonsynonymous point mutation (I49N) variant causes severe disruption of receptor binding, signaling and trafficking in vitro^59^. The mutation occurs in the same highly conserved region of the endofacial portion (i.e., the cytoplasmic end of the TM-1 domain) as is the targeted T2 sgRNA *X. tropicalis mtnr1a* VIL mutation reported here (Fig. 8). Hydrophobic residues on GPCR transmembrane domains, especially TM-1, TM-2, TM-4, and TM-5 are candidates that interface with each other as dimers and/or higher-order oligomeric clusters^60–66^. It is widely recognized that oligomerization of GPCRs often influence ligand binding, G protein coupling, intracellular signaling, and receptor trafficking^67,68^.

Global knockout of MT1 in mice appears to have substantive, yet relatively mild effects on metabolism^3,39,40,69–71^. These observations in knockout mice contrast with the severe rod degeneration phenotype that we report here for the *X. tropicalis mtnr1a* receptor in-frame deletion mutants. These differences make a case for the value of detailed phenotypic analyses of different mutations in the transmembrane domains of melatonin receptors. Such comparisons may lead to greater understanding of the molecular mechanisms by which melatonin receptor signaling contributes to tissue homeostasis. Production of F1 and F2 generations of heterozygous and homozygous *mtnr1a* mutant progeny derived from the F0 founders described here may thus provide unique avenues to gain further insights into molecular mechanisms by which melatonin receptor signaling influences tissue development and homeostasis.

Our observation that a 56-bp deletion mutation (potential knockout; Fig. 7) does not recapitulate the VIL heterozygous mutation (Figs. 3, 4) may guide future efforts to reconcile the dramatic phenotype variances between the *X. tropicalis mtnr1a* VIL non-frameshift deletion and the established mouse MT1 knockout models. The *Xenopus* and mouse models of melatonin receptor disruption therefore have the potential to reinforce and complement one another and to further their applicability to human retinal degenerations. Together, they may provide novel insights on the influence of melatonin receptor circadian signaling in maintaining photoreceptor viability within the context of modern societies that are increasingly untethered from their natural circadian cycles.

## Materials and methods

### sgRNA design and preparation

Three sgRNAs with one or fewer mismatches targeting the *mtnr1a* coding regions were designed using CHOPCHOP (https://chopchop.cbu.uib.no/) and/or CRISPRScan (https://www.crisprscan.org) ^72^. We checked for target specificity by a BLAST search of potential sgRNA targets using GGGenome (https://gggenome.dbcls.jp/). We used the online tool inDelphi, which identifies sgRNA sequences with predictable editing outcomes (https://indelphi.giffordlab.mit.edu/) ^30^ to show that the T2 sgRNA in this study is 27% likely to cause a 9-bp deletion and 18% likely to cause a 12-bp deletion. We also used CRISPRdirect (https://crispr.dbcls.jp/) analysis of the 20-mer T2 sgRNA in this study to show that *no* off-targets occurred in the coding exons elsewhere in the *X. tropicalis* genome^31^. The sgRNAs used in this study were designated as T1, T2, and T3, based on their relative positions downstream of the 5′ start codon. The *mtnr1a* protein (Mel1a) transmembrane domains were determined and plotted using Protter (https://wlab.ethz.ch/protter) ^73^ and GPCRdb (https://gpcrdb.org) ^74^. The nucleotide (nt) sequences used to generate PCR templates for sgRNA synthesis were designed with the protospacer adjacent motif (PAM) site removed and the two nucleotides at the 5′ end of the 20-nt target sequence were altered to GG when necessary in the oligonucleotide forward primer (Supplemental Fig. S1) ^75^. Standard desalted sgRNA forward 60-nt and the 80-nt universal Cas9 reverse oligonucleotide primers^75^ (Supplemental Fig. S1) were custom-synthesized (Sigma). To generate DNA templates for sgRNA synthesis, we used a PCR-based strategy to make linear double-stranded DNA templates for in vitro transcription^76^. The two long, partially overlapping, forward and reverse oligonucleotides were annealed and used as PCR primers with a high fidelity Q5 DNA polymerase (New England BioLabs [NEB]; Supplemental Fig. S13). PCR products were purified using QIAquick (Qiagen) columns, and approximately 300 ng of DNA was used as a template for the SP6 in vitro transcription reaction, using MEGAscript™ SP6 in vitro transcription kits according to manufacturer instructions (Ambion).

### *Xenopus tropicalis* maintenance and embryo microinjections

The *mtnr1a X. tropicalis* F0 founders in this study were generated at the National Xenopus Resource (NXR) of the Marine Biological Laboratory (MBL). They were transported as 2-week old tadpoles to our satellite aquatics facility at the University of Oklahoma Health Sciences Center (OUHSC). They were maintained in accordance with the established animal housing and procedure protocols at the NXR^77,78^, which were approved by the Institutional Animal Care and Use Committees at OUHSC and MBL. Sexually mature adult male and female Nigerian strain *X. tropicalis* were purchased from the NXR (RRID:SCR_013731, https://www.mbl.edu/xenopus), and housed at the OUHSC facility adhering to the protocols described by McNamara et al*.*^77^. Embryos were generated according the procedures described by Wlizla et al*.*^78,79^. One-cell stage embryos were microinjected with 5 nl containing 500 ng of Cas9 protein complexed with 750 pg of both the T1 and T2 sgRNAs, with 0.1% Texas Red dextran. Developing tadpoles were staged according to the *X. laevis* normal table of Nieuwkoop and Faber^80^.

Tadpole siblings that were generated from either sgRNA/Cas9 microinjections or natural matings were raised in identical housing conditions to minimize individual or various environmental influences. Tadpoles were initially maintained in 4-L tanks in an environmental incubator at 27 °C until they were at least 2 weeks old, and then transferred to larger tanks as necessary. They were reared on a 12/12 h light/dark cycle, with an average daytime light exposure of about 900 lx. While in the environmental incubator, animal tanks were rotated daily at feeding time to account for potential differences in their location relative to the lighting source. When they were transferred to larger housing tanks, we continued to monitor the temperature, lighting regime, distance from light source, and water quality for consistency among tanks. All tadpoles were housed at the identical light intensity and duration, tank density, feeding amounts and schedule, and water changes. As much as possible, we used siblings raised together in the same tanks for any particular phenotype analysis.

### gDNA extraction and genotyping assays

Tissues were obtained from tail clips of anesthetized or euthanized tadpoles over four weeks of age and web clips of anesthetized adult frogs, or from euthanized tadpoles less than 15 days old. gDNA was extracted using a Wizard® Genomic DNA Purification Kit (Promega) according to manufacturer’s instructions. gDNA (100–200 ng) from F0 and F1 animals was used as the template in 50 µl PCR reactions with EmeraldAmp® GT PCR Master Mix (Takara) (Supplementary Fig. S13). For T7E1 assays, PCR products were denatured at 95 °C for 3 min and slowly re-annealed to 4 °C at a rate of − 0.01 °C/s. Re-annealed PCR samples were digested with T7E1 (NEB) for 1 h at 37 °C, and then for 2 min at 70 °C to inactivate the enzyme. For RFLP assays, 200–300 ng of *mtnr1a* PCR product was incubated overnight at 37 °C with 1.5 units of the restriction enzyme BseRI (NEB) in NEB 1 × CutSmart® buffer. For direct Sanger sequencing of PCR products, PCR cDNA bands were excised from the gel and purified with EconoSpin® Mini Spin Columns (Epoch Life Science) and the DNA was sequenced with an ABI 3,730 capillary sequencer by the core DNA Sequencing Facility at the Oklahoma Medical Research Foundation (OMRF; Oklahoma City, OK). Sequences were analyzed by trace decomposition (https://tide.nki.nl) to reveal the spectrum of indel sizes and frequencies^81^, and by Poly Peak Parser (https://yosttools.genetics.utah.edu/PolyPeakParser/) to identify indel mutations^82^. To clone PCR products for sequencing, PCR products were inserted into pCR™ 4-TOPO (Invitrogen) cloning vectors in OneShot® TOP10 competent cells according to the manufacturer’s instructions. Plasmid mini-preps from single colonies were sequenced at OMRF using the standard M13 Forward and Reverse primers.

### Histologic analysis and immunohistochemistry (IHC)

Euthanized tadpoles were immersion fixed in 4% paraformaldehyde in PBS for paraffin processing and H&E staining. Double-label IHC was performed on paraffin sections with affinity-purified chicken anti-*X. laevis* Mel1a^35–38^ and rabbit anti-Mel1b antibodies^35,38^. The immunizing peptide for production of the chicken anti-Mel1a receptor was KPDNKPKLKPHDFR, corresponding to the middle region of the third intracellular loop, which is located between TM-5 and TM-6 (Supplementary Fig. S3). Sections were incubated in blocking buffer (2% BSA, 0.2% Triton-X in PBS) and then incubated in the same buffer with 10 μg/ml polyclonal melatonin receptor antibodies overnight at RT. For negative controls, sections were incubated in blocking buffer lacking the primary antibodies or by pre-incubating the primary antibody with an approximate 20-fold molar excess of the immunizing peptide (100 μM). After rinsing in PBS, sections were incubated in combinations of 2 μg/ml of goat anti-rabbit antibody conjugated to AlexaFluor 488 and goat anti-chicken conjugated to AlexaFluor 568 (Molecular Probes). Sections were cover-slipped with ProLong™ Gold antifade reagent with DAPI (Invitrogen). Specimens were viewed by confocal microscopy, using an Olympus FluoView 1,000 laser-scanning confocal microscope. Quantitative measurements of the number of rods and cones per mm of retina were performed with NIS Elements (Nikon) software. Differences in experimental groups were assessed by unpaired two-tailed *t*-tests using GraphPad Prism® to calculate the standard error of the mean and statistical significance between the groups that were analyzed.

## Supplementary information

Supplementary information
