# Supplementary material for: CRISPR/Cas9 mediated mutation of the mtnr1a melatonin receptor gene causes rod photoreceptor degeneration in developing Xenopus tropicalis
Source: Sci Rep. 2020 Aug 13;10:13757. doi: 10.1038/s41598-020-70735-2 (PMC7426423; doi:10.1038/s41598-020-70735-2)
Supplement: Supplementary file 1 — Supplementary information [file 41598_2020_70735_MOESM1_ESM.pdf]

CRISPR/Cas9 mediated mutation of the *mtnr1a* melatonin receptor gene causes rod photoreceptor degeneration in developing *Xenopus tropicalis*

Allan F. Wiechmann, Teryn A. Martin & Marko E. Horb

Xtmtnr1a **sgRNA T1 forward primer:**

ATTTAGGTGACACTATAGAA**GG**GGATGCCAGCCAAGGCAG*[PAM TGG]*GTTTTAGAGCTAGAAATAGC

SP6 promoter **GA replaced** **sgRNA** **PAM sequence omitted** **overlap sequence**

Xtmtnr1a **sgRNA T2 forward primer:**

ATTTAGGTGACACTATAGAA**GG**ACTGAGAGGATAACCAGG*[PAM TGG]*GTTTTAGAGCTAGAAATAGC

AG

Xtmtnr1a **sgRNA T3 forward primer:**

ATTTAGGTGACACTATAGAA**GG**GTTATTGGGAGGATAAAG*[PAM TGG]*GTTTTAGAGCTAGAAATAGC

AG

Universal Cas9 reverse primer:

AAAAGCACCGACTCGGTGCCACTTTTTCAAGTTGATAACGGACTAGCCTTATTTTAACTTGCTATTTCTAGCTCTAAAAC

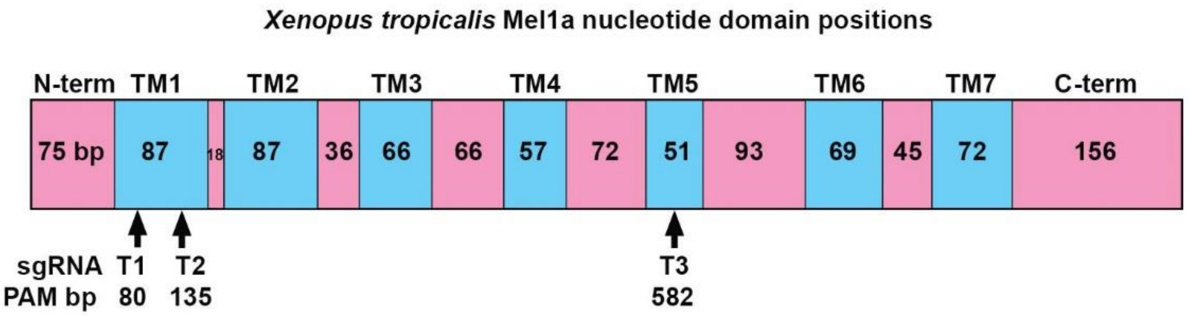

Figure S1. sgRNA template primers and targets of *X. tropicalis mtnr1a* melatonin receptor. ***Upper panel:*** Oligonucleotide PCR primers for DNA template for synthesis of sgRNA are indicated. In the forward primers, the 5′ region has an SP6 promoter binding site, with the downstream **sgRNA target** indicated in **red**. The two 5′ nucleotides of the sgRNAs were changed to **GG** when necessary, as indicated with yellow highlights, with the two original nucleotide sequences positioned immediately below. The three nucleotides of the **PAM sites** located at the 3′ ends of the sgRNA sequences were omitted, and are indicated in **blue**. The 3′ ends of the primers were comprised of “overlap” sequences that are complementary to the reverse sequences of the universal reverse primer (underlined). ***Lower panel:*** The three sgRNAs used in this study were designated as T1, T2, and T3, based on their relative positions downstream of the 5′ start codon. The PAM motif of the T1 sgRNA target was located near the beginning of the first transmembrane domain (80-bp; TM-1), the T2 sgRNA target PAM was near the end of TM-1 (135-bp), with both in the coding region of exon 2. The T3 sgRNA target PAM was located in the middle of the region encoding the TM-5 domain (582-bp), which is in the coding region of exon 3. The numbers in each box represent the number of nucleotides in each domain; transmembrane domains are **blue**, and all others are **red**.

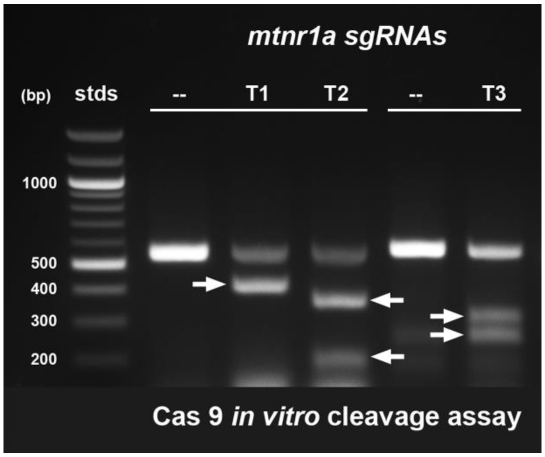

sgRNA	*mtnr1a* target sequence (**PAM** in bold)	Exon target	*In silico* targeting efficiency	*In vitro* targeting effectiveness	Coding region position	Region on protein
T1	**CCA**CTGCCTTGGCTGGCATCCTC	2	51%	69 %	80-102 bp	Beginning of TM-1
T2	**CCT**CCTGGTTATCCTCTCAGTCT	2	75%	83%	135-157 bp	End of TM-1
T3	**CCA**CTTTATCCTCCCAATAACCA	3	48%	43%	388-410 bp	Middle of TM-5

Figure S2. Cas9 *in vitro* cleavage assay with sgRNAs. Genomic DNA from a Nigerian strain wild type (WT) *X. tropicalis* adult was used as the PCR template. DNA PCR products were incubated with sgRNA and Cas9 protein, and then separated on an agarose gel. PCR products of WT gDNA were cleaved into the predicted sizes of DNA fragments (white arrows) when incubated *in vitro* with Cas9 protein (T1, T2, and T3 lanes). The T1 and T2 target sites are in close proximity to each other on exon 2, so the Cas9 cleavage could be detected using the same PCR primer pairs (T12). The T3 target site was in exon 3, so a different set of PCR primers was used to amplify that region. PCR products that were not incubated with the Cas9 enzyme (--) displayed no cleavage bands.

X. tropicalis Mtnr1a scaffold: gi|510884190|ref|NW_004668232.1|

Intron 1: **70 bp**

TAAACCATAATACAATAACATAAAAACTGGTGAGGAATAATTGTAAAATATAACGTTGCAATCTCTAGAA

1 DSP T12 F *(Tm=66.4)*

ATTCCACACGGTGGCGCCCGATTTG**CTTGCACGAAGCTGAAAGGAAT**TTCCCGCTCCCACCACTAGATGT

5′ non-coding

CGCCTGGCGCCTGTCGTTAAGCGGAGCTATAGAAAGCAGCGGCGGGGCCGGGAAGCT**A**TAAAAGTATCCG

2 DSP T12 F *(Tm=64.6)*

ATGTGGCACATGTACATGCAAAGTCAAAACTTGCCGG**CGAGTAAGACATGGCTGCTTC**TCCGTCACTCTC

TTGCGCTCACTTCCATGCATCCGCGTTACGCCGGGCGCAGGGCGCAAAGATGTATGAGAGGAATTGAAAC

3 DSP T12 F *(Tm=62.7)* **4 DSP T12 F** *(Tm=66.3)*

TGCGCGTCCTATGGGGGAAAA**CT*GTCTCAGGGAAGTGCAAGC***GGCACCCAAGAGAAAAATCGGCTGAAAA

Exon 2: **HMA T12 F primer** *(Tm= 61.7)*

ATGAGAGTCAATGGCAGTGCCCTGAACGGCACCTTG**AATATTCCCAAGGAACTGACG**CTGGACCGGCCTC

M R V N G S A L N G T L N I P K E L T L D R P

T1 PAM TM-1 **T2 PAM**

CCTGGGTGC**CCACTGCCTTGGCTGGCATCCTC**ATCTTCACCATCGTGGTGGATATTCTGGGCAA**CCTCCT**

P W V P T A L A G I L I F T I V V D I L G N L L

HMA T12 R primer *(Tm= 61.9)*

GGTTATCCTCTCAGTCTTTAGGAACAGAAAGCTGAGGAACGCA**GGCAAGTACCAGTGTGTGCC**CAACTGG

V I L S V F R N R K L R N A **Intron 2:**

CCAAAGCTCCCCTTCCTGTCTGTGCCATGTACATGTATATGCCAACCCAGCAAGTATGCCCACTGGGTCT

GCCCTGGGACAGTGCTTACCTGCCCACTGGGTCTGCCCTGGGACAGTGCTTACATGCCCACTGGGTCTGC

CCTGGGACAGTGCTTACATGCCCACTGGGTCTGCCCTGGGACAGTGCTTACCTGCCCACTGGGTCTGCCC

1 DSP T12 R *(Tm=66.7)* ***2 DSP T12 R*** *(Tm=63.3)* **4 DSP T12 R** *(Tm=59.4)*

TGGGACAGTGCCCGCATCCTGCTCAA**TTAGCTGGAGGATGGGACATGT*TTGGCCAGTCAGTTTACTCTAG***

GAGAGTGCCTCCATGCCTACCAGTCTGCTCTAGGACATTTGACTGAATGTCTATTCAATTGGCTGCAGGG

TTGTGCTTGCATGGCCACTAAAGTTGCCTGAATGCCCACCCAGGTAGCTGGAGTATGGGGCATGTATGCC

Exon 3: TM-2

AAAAATTAATCATATGTTTTCTTCTTTTTCTTGCTTGTAG**G**GAATATGTTTGTTGTTAGTCTGGCTGTTG

G N M F V V S L A V

CGGACTTGGTGGTCGCCATCTACCCATATCCCTTGGTGCTGACATCAATATTTCACAAAGGATGGA**ATTT**

A D L V V A I Y P Y P L V L T S I F H K G W N L

DSP T3 F primer *(Tm=66.3)* TM-3

GGGATACCTTCACTGCCAGATTAGTGGATTTTTAATGGGAATAAGTGTTATTGGATCCATATTCAACATT

G Y L H C Q I S G F L M G I S V I G S I F N I

GCCGGTGTTGCAGTGAACCGTTATTGTTATATTTGTCATAGCCTCAAATATGACAAGTTGTACAGCGACA

A G V A V N R Y C Y I C H S L K Y D K L Y S D

TM-4 **HMA T3 F primer** *(Tm= 62.1)*

AGAATTCTTTGTTTTATGTCATTCTGATTTG**GGTGCTAACCTTCATTGCC**ATTGTGCCAAATTTATTTGT

K N S L F Y V I L I W V L T F I A I V P N L F V

TGGATCATTACAATACGATGCCAGAATCTACTCGTGTACCTTTACACAGTCCGTTAGCTCAGCATATACC

G S L Q Y D A R I Y S C T F T Q S V S S A Y T

TM-5 **T3 PAM**

ATAGCAGTTGTGTTTTT**CCACTTTATCCTCCCAATAACCA**TAGTAACTTTTTGTTATTTACGTATATGGA

I A V V F F H F I L P I T I V T F C Y L R I W

X. tropicalis Mtnr1a scaffold: gi|510884190|ref|NW_004668232.1|

```
                                     HMA T3 R primer (Tm= 61.8)
TCTTGGTTATTCAGGTAAGACGAAGGGTGAAGCCAGATAACAAACCCAAGCTGAAACCCCATGATTTCAG
I  L  V  I  Q  V  R  R  R  V  K  P  D  N  K  P  K  L  K  P  H  D  F  R
                                   Mel1a antibody peptide sequence

       TM-6
GAACTTTGTAACAATGTTTGTAGTTTTTGTACTGTTTGCAGTCTGCTGGGCACCACTGAACTTCATAGGT
  N  F  V  T  M  F  V  V  F  V  L  F  A  V  C  W  A  P  L  N  F  I  G

                                             DSP T3 R primer (Tm=66.3) TM-7
CTTGCTGTTGCTGTGAACCCGGATACAATTTTACCCAGAATTCCAGAATGGTTGTTTGTTGGCAGCTATT
 L  A  V  A  V  N  P  D  T  I  L  P  R  I  P  E  W  L  F  V  G  S  Y

ACATGGCATATTTTAACAGTTGCCTTAATGCTATCATTTATGGTCTCTTAAACCAAAATTTTAGACGAGA
Y  M  A  Y  F  N  S  C  L  N  A  I  I  Y  G  L  L  N  Q  N  F  R  R  E

GTACAAAAGAATTATTATCTCCATTTGTACAGCAAAGGTTTTCTTTCAAGAAAGTTCTAATGACGGGGTA
  Y  K  R  I  I  I  S  I  C  T  A  K  V  F  F  Q  E  S  S  N  D  G  V

                                                                    Stop
GAGAAAATGAAAAGCAAACCATCTCCAATGCTAACAAACAATAATCTAGTAAAAGTTGATTCAGTTTAGG
 E  K  M  K  S  K  P  S  P  M  L  T  N  N  N  L  V  K  V  D  S  V  *

3′ non-coding:
GGAAAAAAGGAATTCTTGCAAATGATAGTATACAGGTATGGGACCTGTTATCCAGAACGCTTGGGACCTG
```

Figure S3. *Xen*opus (Silurana) tro*picalis mtnr1a* genomic DNA sequence and genotyping primer locations. The gDNA sequence was obtained from the Xenbase *X. tropicalis* Nigerian 7.1 Genome scaffold database (http://www.xenbase.org/entry/). **Exon/intron** boundaries are indicated in bold above or below the nucleotide sequence. **Primer pairs** *(with Tm=°C)* used for PCR are indicated in matching colors or shading and are underlined and in bold letters. The **PAM** sequences are indicated in bold yellow highlights and the **sgRNA target sequences** are indicated in bold and underlined. **Amino acids** in the exons appear below their corresponding codons, and are indicated in red. Protein transmembrane domains (**TM-1 - TM-7**) are shaded gray with red lettering. The non-coding 5′ and 3′ ends of the *mtnr1a* sequence are highlighted in yellow. The 13-amino acid sequence in the third intracytoplasmic loop corresponding to the *X. laevis* Mel1a receptor antibody immunizing peptide (KPDNKPKLKPHDFR) is underlined in red.

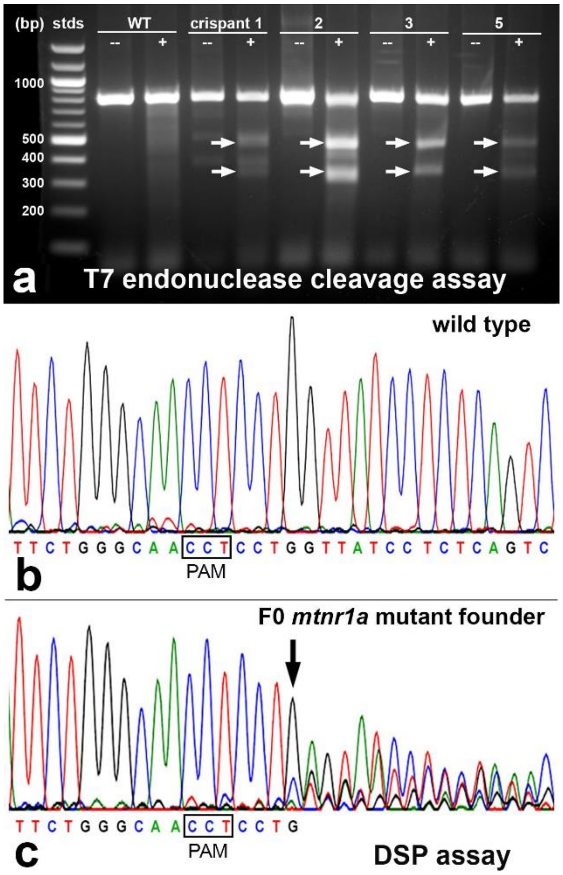

Figure S4. Genotyping assays identify adult F0 *X. tropicalis* sibling crispants with *mtnr1a* gene VIL mutations. **(a)** Representative T7 endonuclease I (T7EI) mismatch assay identifies mosaic F0 animals that express T2 *mtnr1a* indels. Denatured/reannealed amplicons are cleaved by T7E1 at the site of mismatches due to mutations in one strand, which generate DNA cleavage fragments on agarose gels. The crispants in this assay are assigned the same numbers as in supplementary figures S5 and S6, in which crispant #2 is the male founder of the F1 *mtnr1a* mutant progeny. All of the crispants shown here are mutants, based on the presence of PCR cleavage fragments when incubated with T7EI (+; arrows). None of the samples are cleaved in the absence of the T7E1 enzyme (--), and the DNA of normal homozygous (WT) progeny is not cleaved by T7EI (+). The image was enhanced digitally in Photoshop for contrast and brightness, and to mask some undesirable flecks of ethidium bromide precipitate. **(b)** Direct PCR Sanger sequencing chromatogram of WT progeny displays prominent peaks for all bases in the T2 sgRNA target area. The T2 PAM sequence (CCT) is boxed in black. **(c)** Direct PCR sequencing chromatogram of an F0 mosaic founder (crispant #2 in supplementary figures 5 and 6) shows pronounced sequence decomposition beginning 4-bp downstream of the T2 PAM site (arrow), representing the presence of a mixture of WT and mutant *mtnr1a* T2 target cDNAs from the gDNA of the mosaic tissue sample.

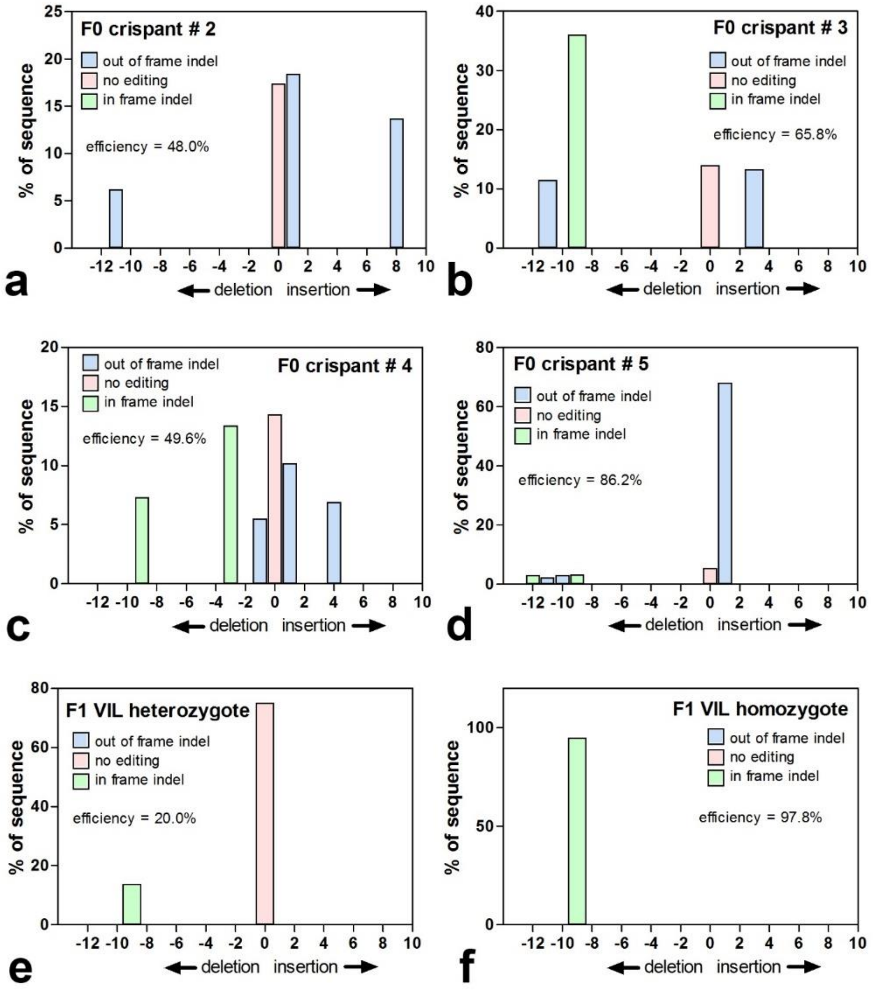

Figure S5. Comparison of the editing efficiency of the *mtnr1a* T2 sgRNA in F0 mutant animals. Trace decomposition yields the spectrum of indel size and frequency occurring within a 0 to 12-bp indel size range. **(a)** The F0 crispant #2, which is the male founder of all of the F1 progeny described in this study, does not display a 9-bp deletion in this TIDE analysis of PCR products from gDNA extracted from a pair of web clips. **(b-d)** F0 sibling crispants #3-5 all display a 9-bp deletion at various frequencies, with crispant #3 having the highest frequency. **(e)** For comparison, PCR of web clip gDNA from an F1 heterozygous VIL mutant frog displays the expected indels only at 0 and -9-bp. **(f)** A sibling F1 homozygous VIL mutant frog displays a -9-bp indel only, as anticipated. TIDE analysis was performed using http://tide.nki.nl [81].

mtnr1a T2 F0 sibling clones: ⬇ end of exon 2

```
 V  V  D  I  L  G  N  L  L  V  I  L  S  V  F  R  N  R  K  L  R  N  A       CDS
GTGGTGGATATTCTGGGCAACCTCCTGGTTATCCTCTCAGTCTTTAGGAACAGAAAGCTGAGGAACGCA     Wild-type
```

Crispant #1:

```
GTGGTGGATATTCTGGGCAACCTC------------TCAGTCTTTAGGAACAGAAAGCTGAGGAACGCA     (Δ12)[×9]-LVIL
GTGGTGGATATTCTGGGCAA-----------TCCTCTCAGTCTTTAGGAACAGAAAGCTGAGGAACGCA     (Δ11)     FS*
GTGGTGGATATTCTGGGCAACCTCCTctctGgGCaaTATCCTCTCAGTCTTTAGGAACAGAAAGCTGAG     (+7)[×2]  FS
```

Crispant #2:

```
GTGGTGGATATTCTGGGCAACC-----------------------------AGAAAGCTGAGGAACGCA     (Δ29)[×11]  FS
GTGGTGGATATTCTGGGCAACCTCCcaacTGagaGTTATCCTCTCAGTCTTTAGGAACAGAAAGCTGAG     (+7)[×4]    FS
GTGGTGGATATTCTGGGCAA----------------------------------AAGCTGAGGAACGCA     (Δ34)[×3]   FS
GTGGTGGATATTCTGGGCAACCTC------------TCAGTCTTTAGGAACAGAAAGCTGAGGAACGCA     (Δ12)[×3]-LVIL
GTGGTGGATATTCTGGGCAAC------------------------------AGAAAGCTGAGGAACGCA     (Δ30)[×2]
GTGGTGGATATTCTGGGCAA-----------TCCTCTCAGTCTTTAGGAACAGAAAGCTGAGGAACGCA     (Δ11)  FS*
GTGGTGGATATTCTGGGCAACCTCC------------CAGTCTTTAGGAACAGAAAGCTGAGGAACGCA     (Δ12)-VILS/L>P
GTGGTGGATATTCTGGGCAACCTCCTGGATATTCTGGGC---------AACAGAAAGCTGAGGAACGCA     (Δ9)   sub6-VFR
GTGGTGGATATTNTGGGCAAC-----------------------------CAGAAAGCTGAGGAACGCA     (Δ29)  FS
GTGGTGGATATTCTGGGCA------------------------------CCATAAAGCTTAGGAACGCA     (Δ30)  sub2
GTGGTGGATATTCT-------TTCCT------------------------CAGAAAGCTGAGGAACGCA     (Δ31)  sub1 FS
GTGGTGGATATTCTGGGCAACCTCtcagtcCTtaGGTTATCCTCTCAGTCTTTAGGAACAGAAAGCTGA     (+8)   FS
```

Crispant #3:

```
GTGGTGGATATTCTGGGCAACCTCCT-----------CAGg-TTTAGGAACAGAAAGCTGAGGAACGCA     (Δ12)-VILS/V>R[×2]
GTGGTGGATATTCTGGGCAACCTCCT-----------CAGTCTTTAGGAACAGAAAGCTGAGGAACGCA     (Δ11)  FS**
GTGGTGGATATTCTGGGCAACCTCCTGGgTTATCCTCTCAGTCTTTAGGAACAGAAAGCTGAGGAACG     (+1)   FS
GTGGTGGATATTCTGGGCAACCTCtCAGTcTTATCCTCTCAGTCTTTAGGAACAGAAAGCTGAGGAACG     (+2)   FS
GTGGTGGATATTCTGGGCAACCTCCaacTTATCCTCTCAGTCTTTAGGAACAGAAAGCTGAGGAACGCA     (+3)   LV>QL
```

Crispant #4:

```
GTGGTGGATATTCTGGGCAACCTCCTGGgTTATCCTCTCAGTCTTTAGGAACAGAAAGCTGAGGAACGC     (+1)  FS***[×3]
GTGGTGGATATTCTGGGCAACCTC------------TCAGTCTTTAGGAACAGAAAGCTGAGGAACGCA     (Δ12)-LVIL
GTGGTGGATATTCTGGGCAACCTCCT-----------CAGTCTTTAGGAACAGAAAGCTGAGGAACGCA     (Δ11)FS**
GTGGTGGATATTCTGGGCAACCTCCTGcataGTTTATCCTCTCAGTCTTTAGGAACAGAAAGCTGAGGA     (+4)  FS
GTGGTGGATATTCTGGGCAACCTCCTGGTTATCCTCTCGGTCTTTAGGAACAGAAAGCTGAGGAACGCA           sub1
```

Crispant #5:

```
GTGGTGGATATTCTGGGCAACCTCCTGGgTTATCCTCTCAGTCTTTAGGAACAGAAAGCTGAGGAACGC     (+1)   FS***[×3]
GTGGTGGATATTCTGGGCAACCTCCT-----------CAGTCTTTAGGAACAGAAAGCTGAGGAACGCA     (Δ11)  FS**
GTGGTGGATATTCTGGGCAACCTCTCA-GTTATCCTCTCAGTCTTTAGGAACAGAAAGCTGAGGAACGC     (Δ1)   sub3 FS
GTGGTGGATATTCTGGGCAACCTCCAGGTTGCCCAGAACCTGGACT----------GCTGAGGAACGCA     (Δ10)  sub14 FS
```

CCT = PAM T2 sequence
CCTGGTTATCCTCTCAGTCT=T2 target
------ = deletion (Δ)
green = insertion (+)
Blue = substitution (sub)
FS = frameshift mutation
[×] = # of F0 clones
CDS = amino acid coding sequence

TAG/TGA = premature stop codon
-LVIL = LVIL 12-bp deletion
-VILS/L>P = VILS 12-bp deletion/L to P substitution
-VILS/V>R = VILS 12-bp deletion/V to R substitution
–VFR = VFR 9-bp deletion
LV>QL = LV to QL substitution
# of asterisks = FS mutation match in another animal

Figure S6. Sanger sequencing of plasmid clones of five adult F0 siblings with *mtnr1a* T2 mutations. Crispant #2 is the male founder of all F1 animals in this study. All crispants exhibit indels that are common to two or more animals. The black arrow (⬇) indicates the 3′ end of exon 2. Since the T2 sgRNA target was near the end of exon 2, not all frameshift (FS) mutations resulted in premature stop codons in exon 2.

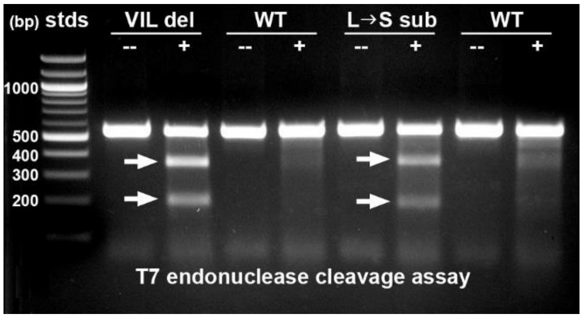

Figure S7. Identification of heterozygous F1 animals with CRISPR/Cas9 indel mutations of the *mtnr1a* gene. Representative T7E1 assay of two heterozygous mutant *mtnr1a* F1 and two F1 WT sibling tadpoles. PCR products amplified from gDNA extracted from F1 progeny were denatured to separate the cDNA strands, then re-annealed slowly to promote formation of mismatched WT/mutant double-stranded cDNA. Mismatched sites of re-annealed WT and mutant DNA strands in heterozygous tadpoles are cleaved by T7E1 (+), resulting in two cleavage products (arrows) in a tadpole with the 9-bp (non-frameshift VIL) *mtnr1a* deletion, and for comparison, in another tadpole with a 3-bp substitution (predicted amino acid change is L→S; see supplementary figure S7). The DNA of normal homozygous (WT) progeny is not cleaved by T7E1 (+), and none of the samples are cleaved in the absence of the enzyme (--).

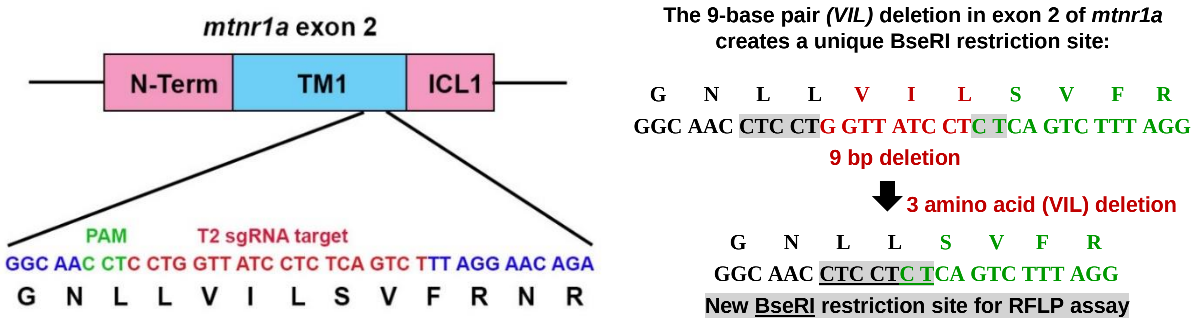

Figure S8. A novel restriction fragment length polymorphism *(RFLP)* assay uniquely identifies VIL mutants. ***Left panel:*** Schematic representation of exon 2 of the *mtnr1a* gene, with the TM-1 domain flanked by the amino-terminus (N-Term) and first intracellular loop (ICL-1) domains. The nucleotide sequence targeted by the **T2 sgRNA** at the lower area of the panel is indicated in **red**, with the concomitant amino acid sequence below. The protospacer-adjacent motif (**PAM**) sequence, which directs Cas9 targeting, is indicated in **green**. Some flanking nucleotide sequence is indicated in **blue**. ***Right panel:*** The VIL deletion merges the upstream **CTCCT** sequence with the downstream **CT** sequence to create a unique BseRI restriction site.

***mtnr1a*T12 F1 progeny clones:** ⬇ end of exon 2

Sequence		
V V D I L G N L L V I L S V F R N R K L R N A	CDS	
GTGGTGGATATTCTGGGCAA<u>CCT</u>CCTGGTTATCCTCTCAGTCTTTAGGAACAGAAAGCTGAGGAACGCA	Wild-type	
GTGGTGGATATTCTGGGCAA<u>CCT</u>CCT---------CTCAGTCTTTAGGAACAGAAAGCTGAGGAACGCA	(Δ9) [×12]	–VIL
GTGGTGGATATTCCGGGCAA<u>CCT</u>CTCAGTTATCCTCTCAGTCTTTAGGAACAGAAAGCTGAGGAACGCA	sub3, [×8]	L>S
GTGGTGGATATTCTGGGCAG<u>CC</u>---------TCCTCTCAGTCTTTAGGAACAGAAAGCTGAGGAACGCA	(Δ9), sub1	–LVI, N>S
GTGGTGGATATTCTGGGCAA<u>CCT</u>CCTGGTTATCCCCTCAGTCTTTAGGAACAGAAAGCTGAGGAACGCA	sub1	L>P

Legend:

<u>CCT</u> = PAM T2 sequence
CCTGGTTATCCTCTCAGTCT = target site
––– = deletion (Δ)
Blue = substitution (sub)
[× # of different F1 animals]

–VIL = VIL 3-aa (9-bp) deletion
L>S = leucine to serine (3-bp) substitution (missense)
–LVI, N>S = LVI 3-aa (9-bp) deletion, and arginine to serine (1-bp) substitution
L>P = leucine to proline (1-bp) substitution (missense)
FS = frameshift mutation (nonsense)

Figure S9: Sequence alignment of plasmid clones of *mtnr1a* F1 progeny. Sanger sequencing of 22 plasmid clones of F1 with *mtnr1a* T2 progeny revealed that 54.5% (12/22) of clones from mutant animals expressed the VIL deletion. No frameshift mutations were observed in these samples.

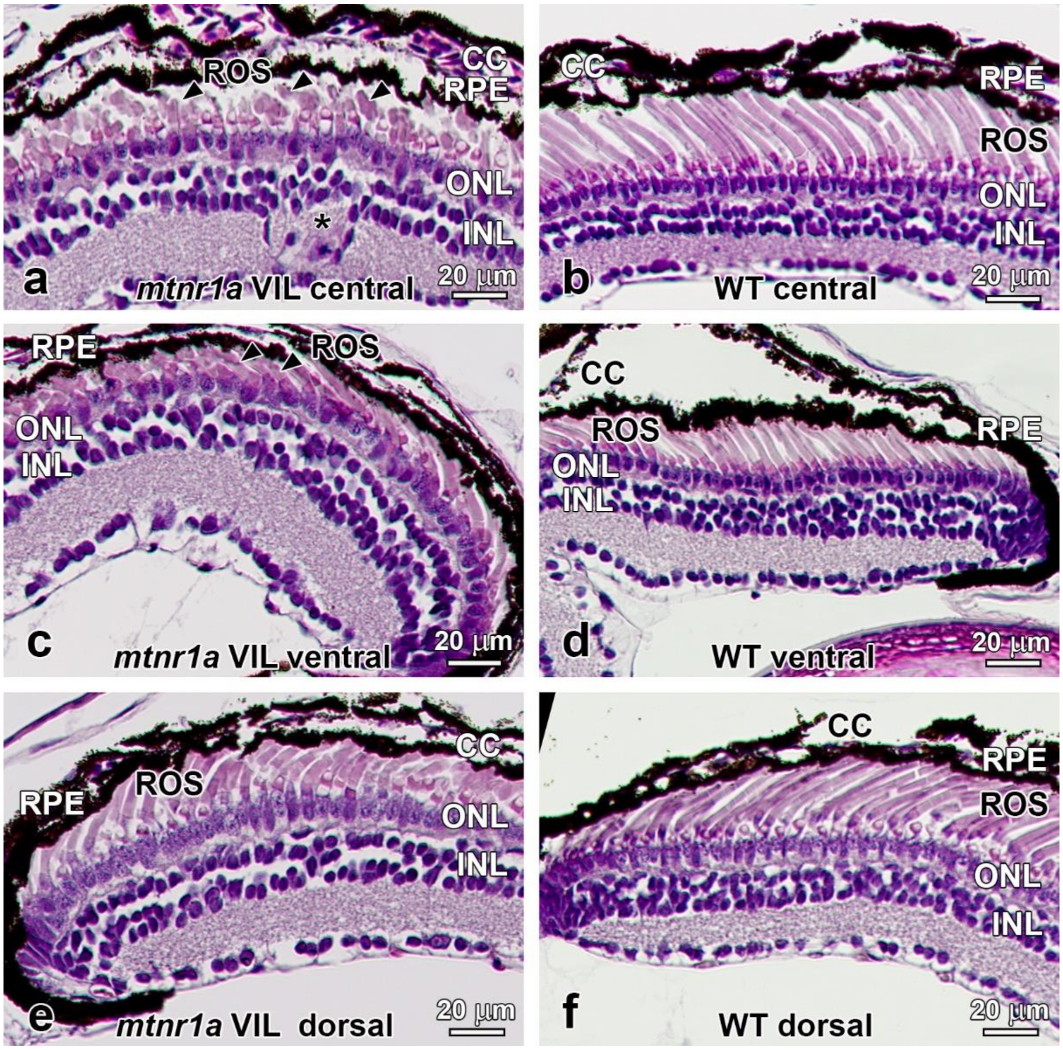

Figure S10: Rod photoreceptor degeneration is more severe in the central vs. peripheral regions of stage 54-56 (48-day) *mtnr1a* VIL mutants. H&E sections of 48-day *mtnr1a* VIL heterozygous mutant (a,c,e) and WT (b,d,f) sibling tadpole retinas. **(a,b)** ROS dystrophy (black arrowheads) is substantial in the central region of the *mtnr1a* VIL heterozygous mutant retina (a), whereas ROS morphology appears normal in central retinas of WT sibling tadpoles (b). The VIL mutant inner retina shows some atypical clusters of cells (a; asterisk). **(c,d)** In the ventral region, ROS were consistently shorter in *mtnr1a* mutants (c) than in WT (d) sibling tadpole retinas. **(e,f)** The ROS in the dorsal peripheral region of the *mtnr1a* VIL mutant retina (e) also appeared to be shorter than in WT sibling dorsal retinas (f), but the difference was less pronounced than in the ventral peripheral retina. CC; choriocapillaris, RPE; retinal pigment epithelium, ROS; rod outer segments, ONL; outer nuclear layer, INL; inner nuclear layer. Magnification bars = 20 µm.

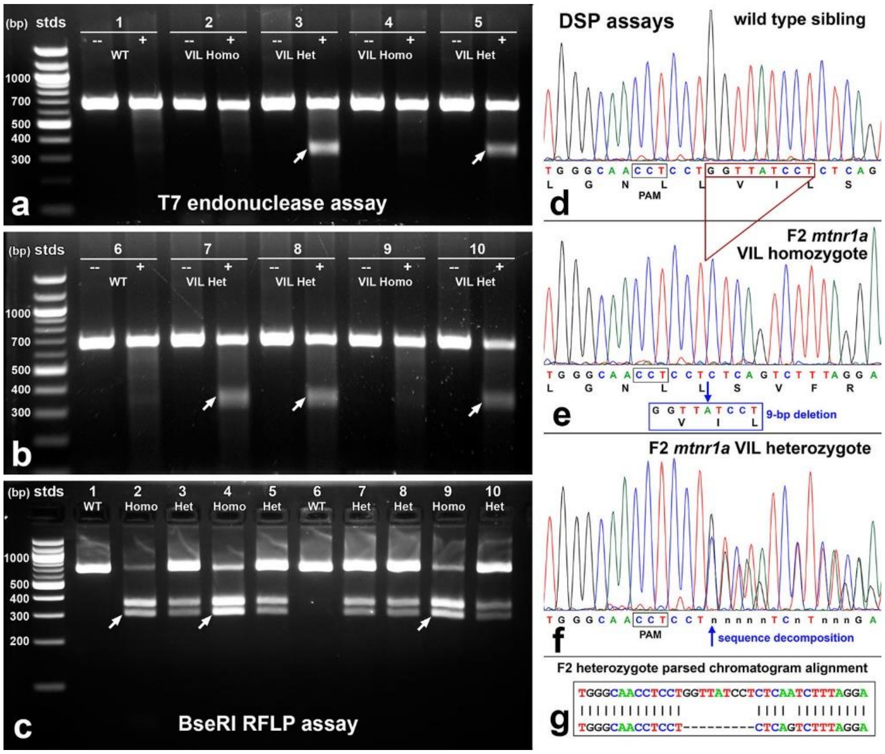

Figure S11. Identification of F2 heterozygous and homozygous *mtnr1a* VIL mutant tadpoles. (a,b) T7E1 assay of ten random sibling F2 tadpole progeny from a crossing of two heterozygous *mtnr1a* VIL mutant frogs. PCR products amplified from gDNA were denatured and re-annealed slowly to promote formation of mismatched WT/mutant double-stranded cDNA. Mismatched sites are cleaved by T7E1 (+; arrows) in VIL *mtnr1a* heterozygotes. The DNA of WT (homozygous; lanes 1 and 6) or homozygous VIL mutant (lanes 2, 4, and 9) siblings is not cleaved by T7E1 (+), and none of the samples are cleaved in the absence of the enzyme (--). In this assay, tadpoles 3, 5, 7, 8, and 10 are identified as VIL heterozygotes (arrows). **(c)** BseRI RFLP analysis of the same PCR products as in (a,b). *Mtnr1a* VIL heterozygotes (lanes 3, 5, 7, 8, and 10) and homozygotes (lanes 2, 4, and 9) display two predicted cleavage bands (arrows), whereas WT DNA (lanes 1 and 6) is not cleaved. Note that the amount of uncleaved PCR template of the VIL homozygous mutants is much lower than in the homozygotes and WT, whereas the amount of cleavage product is higher than the others, illustrating that both alleles in homozygous mutants are cleaved in this assay. Illustration of the creation of a BseRI site by the VIL deletion is in Supplementary Figure S8. **(d)** Direct PCR Sanger sequencing chromatogram (DSP assays) of F1 WT progeny display prominent peaks for all bases in the T2 sgRNA target area. The T2 PAM sequence (CCT) is boxed in **black**, and the downstream 9-bp sequence of the *mtnr1a* VIL deletion mutation is boxed in **red**. The corresponding amino acid sequence is shown below the nucleotide sequence. **(e)** The boxed 9-nucleotide sequence in (d) is missing in the F2 *mtnr1a* VIL chromatogram, indicating that the 9-bp deletion is present on both alleles. The deleted sequence is boxed in **blue** below the chromatogram. **(f)** In F2 mutants that heterozygous for the *mtnr1a* 9-bp (VIL) deletion, the trace decomposes (*i.e.*, mixed base calls) at the predicted target site 4-bp downstream of the T2 PAM site, and continues to the end of the trace, as expected for a heterozygous indel mutation. The decomposed sequence represents the overlapping peaks of the *mtnr1a* WT and VIL alleles. **(g)** Partial view of alignments from Poly Peak Parser (http://yosttools.genetics.utah.edu/PolyPeakParser/) [82] output displaying the heterozygous 9-bp *mtnr1a* VIL deletion mutation. The WT allele is the top line and the mutant allele is the bottom line.

Exon 2 **T2 PAM** & sgRNA target

```
 I  L  I  F  T  I  V  V  D  I  L  G  N  L  L  V  I  L  S  V
ATCCTCATCTTCACCATCGTGGTGGATATTCTGGGCAACCTCCTGGTTATCCTCTCAGTC
|||||||||||||||||||||||||||||||||||||||||||||
ATCCTCATCTTCACCATCGTGGTGGATATTCTGGGCAACCTCCTG---------------
 I  L  I  F  T  I  V  V  D  I  L  G  N  L  L
```

Exon2/Intron 2 boundary

```
 F  R  N  R  K  L  R  N  A  G  K  Y  Q  C  V  P  N  W  P  K
TTTAGGAACAGAAAGCTGAGGAACGCAGGCAAGTACCAGTGTGTGCCCAACTGGCCAAAG
                                         |||||||||||||||||||
-----------------------------------------TGTGCCCAACTGGCCAAAG
                                          C  A  Q  L  A  K
```

56-bp deletion frameshift mutation

```
 L  P  F  L  S  V  P  C  T  C  I  C  Q  P  S  K  Y  A  H  W
CTCCCCTTCCTGTCTGTGCCATGTACATGTATATGCCAACCCAGCAAGTATGCCCACTGG
||||||||||||||||||||||||||||||||||||||||||||||||||||||||||||
CTCCCCTTCCTGTCTGTGCCATGTACATGTATATGCCAACCCAGCAAGTATGCCCACTGG
A  P  L  P  V  C  A  M  Y  M  Y  M  P  T  Q  Q  V  C  P  L

 V  C  P  G  T  V  L  T  C  P  L  G  L  P  W  D  S  A  Y  M
GTCTGCCCTGGGACAGTGCTTACCTGCCCACTGGGTCTGCCCTGGGACAGTGCTTACATG
 |||||||||||||||||||||||||||||||||||||||||||||||||||||||||||
GTCTGCCCTGGGACAGTGCTTACCTGCCCACTGGGTCTGCCCTGGGACAGTGCTTACATG
G  L  P  W  D  S  A  Y  L  P  T  G  S  A  L  G  Q  C  L  H

 P  T  G  S  A  L  G  Q  C  L  H  A  H  W  V  C  P  G  T  V
CCCACTGGGTCTGCCCTGGGACAGTGCTTACATGCCCACTGGGTCTGCCCTGGGACAGTG
 |||||||||||||||||||||||||||||||||||||||||||||||||||||||||||
CCCACTGGGTCTGCCCTGGGACAGTGCTTACATGCCCACTGGGTCTGCCCTGGGACAGTG
A  H  W  V  C  P  G  T  V  L  T  C  P  L  G  L  P  W  D  S
```

Figure S12. Alignment of WT and F1 *X. tropicalis mtnr1a* heterozygous 56-bp deletion mutant. The upper sequence is WT with hypothetical amino acids in the non-coding intron corresponding to the codon below indicated in **blue**. The mutant is the lower sequence, with the hypothetical amino acid sequence *centered* over the corresponding codon indicated in **red**. The T2 sgRNA target sequence is underlined with the PAM sequence underlined in **bold**. The 56-bp deletion occurs at the boundary of exon 2 and intron 2 (Exon 2/Intron 2 boundary), and is indicated by red dashes (**---**). Since 56 is not a multiple of three, the deletion causes a hypothetical shift in the reading frame. Note that the amino acids indicated in blue or red downstream of the mutation are not expected to be expressed, and are shown only to illustrate the misalignment of nucleotides due to the putative frameshift. Since the exon/intron splice site is skipped in the mutant due to the 56-bp deletion, the non-coding region downstream of the deletion could theoretically encode aberrant protein, indicated in **red**. No stop codons were observed in the mutant sequence downstream of the deletion.

PCR to make DNA template for sgRNA synthesis:

PCR components:

1 µl forward 100 µM primer
1 µl reverse 100 µM primer
2 µl 10 mM dNTPs
0.5 µl of Q5 DNA polymerase (New England BioLabs)
10 µl Q5 buffer
85.5 µl
100 µl total volume

PCR conditions:

10 cycles of 98° 10 sec, 62° 20 sec, and 72° 20 sec
25 cycles of 98° 10 sec, 72° 30 sec
5 min 72°

PCR to make DNA template for *in vitro* Cas9 cleavage assays:

300 ng WT gDNA
0.2 µM forward primer
0.2 µM reverse primer
2X EmeraldAmp® GT PCR Master Mix (Takara)

Primer sequence for T1 and T2 sgRNA templates:

Forward primer: 5′-CTGTCTCAGGGAAGTGCAAGC-3′
Reverse primer: 3′-CTAGTAAACTCACTGGCCAA-5′
540-bp amplicon

Primer sequence for T3 sgRNA template:

Forward primer: 5′-ATTTGGGATACCTTCACTGCCA-3′
Reverse primer: 3′-CTGCCAACAAACAACCATTCTG-5′
559-bp amplicon

PCR conditions:

98° 1 min
35 cycles of: 98° 10 sec, 62° (T12) or 65° (T3) 30 sec, 72° 1 min
72° for 5 min

PCR to make *mtnr1a* cDNA from genomic DNA:

100-200 ng gDNA
0.2 µM forward primer (see Figure S3 for locations and T_M)
0.2 µM reverse primer (see Figure S3 for locations and T_M)
2X EmeraldAmp® GT PCR Master Mix (Takara)

Primer sequence for T1 and T2 mtnr1a cDNA from gDNA template:
Forward primer: 5′- CTGTCTCAGGGAAGTGCAAGC -3′
Reverse primer: 3′- CTAGAGTAAACTGACTGGCCAA -5′
542-bp amplicon

PCR conditions:
98°C 1 min
35 cycles of: 98°C 10s, 62°C (T12) or 65°C (T3) 30s, 72°C 1 min
72°C for 5 min

Figure S13: PCR conditions for sgRNA template synthesis, Cas9 cleavage assays, and PCR amplification of gDNA.
